# Structures control gold mineralization in the western Allaqi shear belt of Egypt from high-precision geophysical and remote sensing datasets

**DOI:** 10.1038/s41598-025-27310-4

**Published:** 2025-12-04

**Authors:** Ahmed M. Eldosouky, Mohamed A. Abd El‑Wahed, Mohamed Abd El Monsef, Mohamed Attia

**Affiliations:** 1 Department of Geology, Faculty of Science, Suez University, P.O. Box: 43221, Suez, Egypt; 2 Geology Department, Faculty of Science, Tanta University, P.O. Box: 31527, Tanta, Egypt; 3 State Key Laboratory of Continental Dynamics, Department of Geology, Northwest University, Xi’an, 710069 China; 4 Geology Department, Faculty of Science, Kafr El Sheikh University, P.O. Box: 33511, Kafr El Sheikh, Egypt

**Keywords:** Geophysics, Edge Detection, Western Allaqi shear belt, Remote Sensing, Tectonics, Gold Mineralization, Eastern Desert, Geophysics, Structural geology

## Abstract

This study employs Remote Sensing, advanced aeromagnetic edge detectors, and fieldwork to map structural features influencing mineralization in Egypt’s western Allaqi shear belt. Four edge detectors were tested on synthetic models; the hyperbolic tangent function and a novel edge detector were most effective at delineating edges and lineaments. These were applied to RTP aeromagnetic data to identify shallow and deep structures. The belt features an E-W striking, steeply north-dipping foliation (S1), overturned and recumbent folds (F1), and shear zones from serpentinite emplacement over volcaniclastic metasediments and metavolcanics. Thrust planes have been deformed by D2 folds with west-plunging hinges and steeply dipping cleavages oriented NE and ENE. D3 deformation turned east–west and northwest-trending folds into north-trending ones due to shearing, giving the region a N-trending fold pattern. D4 caused northeast-trending folds from shear zones; D5 formed faults in ENE-WSW, NE-SW, and N-S directions. D4 structures control gold deposits in WASB, with S4 foliation, NE-trending folds, and shearing. Haimur Au deposits align with main shearing; Um Ashira Au intersects rocks; Hariari Au trends ENE. Landsat-8 bands identified minerals like ferrous and ferric oxides, hydroxyl alterations, and chlorite zones. Higher lineament density links to increased fracturing and mineralization. Two maps highlight ore-rich areas. Combining data improves understanding of tectonic evolution and mineralization, enhancing exploration in complex terrains.

## Introduction

**Fig. 1:**
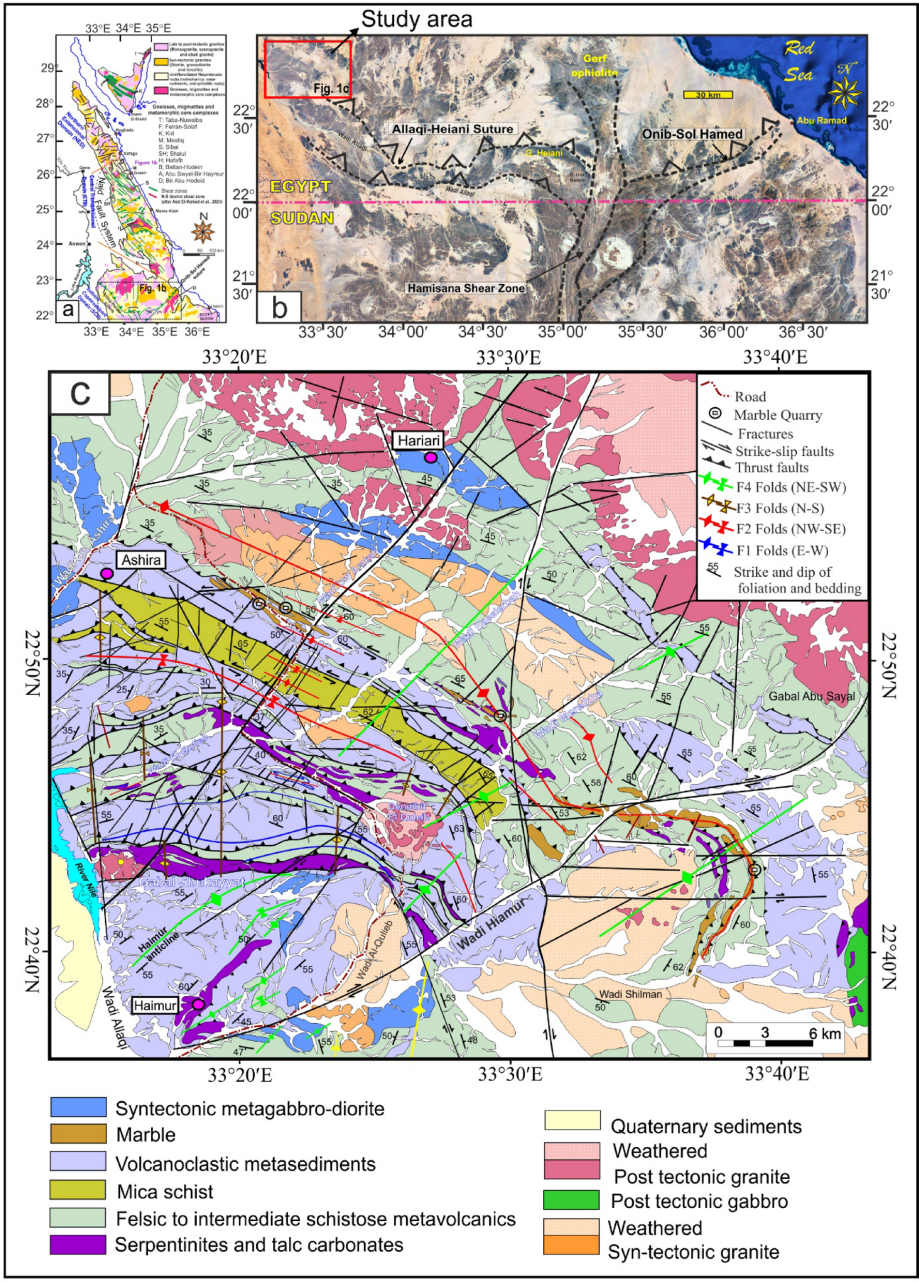
(**a**) Simplified geological map of the Eastern Desert of Egypt and Sinai showing the Najd fault zone and major structures in the Egyptian Nubian Shield (modified from^13–15^, (**b**) Landsat image showing location of Allaqi-Heiani Suture and the study area, and (**c**) Geological map of Western Allaqi shear belt (after Abd El-Wahed et al.^16^ and EGSMA,^17^). Map created by ArcGIS Desktop v 10.7.1. https://www.esri.com/en-us/arcgis/products/arcgis-desktop/overview. Image processing performed by ENVI software v. 5.3. https:// www.l3harrisgeospatial.com/software-technology/ENVI.

Tectonic and structural controls are among the considerable elements governing mineralization, particularly in shear belt-hosted deposits like the western Allaqi shear belt, South Eastern Desert (SED) of Egypt (Fig. 1a and b). The relations of local and regional deformation, magmatic-hydrothermal processes, and structure reactivation dictate the spatial distribution of ore deposits ^1–6^. Understanding these geological and structural controls is crucial for improving exploration models and recognizing unexplored probable mineralized sites^5,7–12^.

Refinements in remote sensing (RS) and geophysical strategies have advanced structural mapping, allowing the delineation of deep-seated and shallow structures that are not consistently observable at the surface. Aeromagnetic data enhanced by employing high-precision edge detection (ED) methods can reveal intrusive contacts/boundaries, shear zones/belts, and structures networks with remarkable detail^18–26^.

ED is widely utilized in detecting ore deposits, faults, contacts, and dikes from potential (magnetic and gravity) fields^27–32^F. Many ED methods have been designed to enable interpretation^33–41^). Alvandi et al.^42^ presented filters using the total horizontal gradient (THG) derivatives. Alvandi and Ardestani^43^ proposed using the Gompertz function to delineate the boundaries of sources. Nasuti et al.^44^ introduced an improved filter (STDR) for better estimating the edges. In addition, some high-resolution approaches were introduced to map structural boundaries^45,46^. Many authors used aeromagnetic data interpretation and integration of geological and geophysical analyses to delineate potential mineral zones and controlling structures^47–54^.

Recent innovations in remote sensing technology have significantly improved the assessment of structural and geological features. Multi-sensor satellite imagery, especially using VNIR and SWIR spectral data, serves as a valuable resource for geological research and the extraction of lineaments such as faults, fractures, and folds^13,55–60^. The spectral data from satellite imagery used in mineral exploration covers wavelengths from the visible spectrum to the infrared range. Hydrothermal alteration is often associated with hydrothermal mineral deposits, making identifying and mapping hydrothermal alteration zones a key goal of mineral exploration activities that use remote sensing data^13,57,58,61^. Many minerals linked to hydrothermal alteration show distinct shortwave infrared (SWIR) spectrum features. This trait makes the Advanced Spaceborne Thermal Emission and Reflection Radiometer (ASTER) and the Landsat-8 Operational Land Imager (OLI) sensors valuable and cost-effective tools for mineral mapping, particularly in arid regions.

The main goal of our investigation is to analyze the tectono-structural framework controlling mineralization in the western Allaqi shear belt, SED of Egypt (Fig. 1a and b). High-precision structural delineations at various depths using accurate EDs will be employed to map both deep-seated and shallow controls on mineralization. To validate aeromagnetic interpretations, RS and field structural data will be analyzed to map rock fabrics, deformation structures, and alteration zones. The structural interpretation of mineral deposits in the western Allaqi shear belt will connect structures, mineralization patterns, and shear deformation to regional tectonics. Our analysis will provide new insights into the evolution of the west Allaqi shear belt, magmatic-hydrothermal processes, and how structures can influence gold mineralization.

## Geological setting

The western Allaqi shear belt is located in the western section of the Wadi Allaqi area within the southeastern desert of Egypt, in proximity to Nasser Lake and the Nile Valley (Fig. 1a and 1b), approximately 220 km from Aswan city via an asphalt road in Upper Egypt (Fig. 1a and 1b). The western Allaqi shear belt (Fig. 1c) is composed of an ophiolitic assemblage that includes serpentinite, listwenite, talc carbonate, metagabbro, and metabasalt^16^. Listwenite is produced due to the alteration of serpentinized peridotite, a process influenced by carbonation and/or silicification related to hydrothermal activity^62–67^. The island arc rocks include metavolcanics and volcanoclastic metasediments (Fig. 1c). The metasedimentary components include mica schist, metasiltstone, metagraywacke, and quartz feldspathic schist, all exhibiting a calc-alkaline affinity^68^. The metavolcanics consist of highly sheared metaandesite, metadacite, and pyroclastic materials formed during an early immature phase of island arc tectonic environment^66,69,70^.

**Fig. 2:**
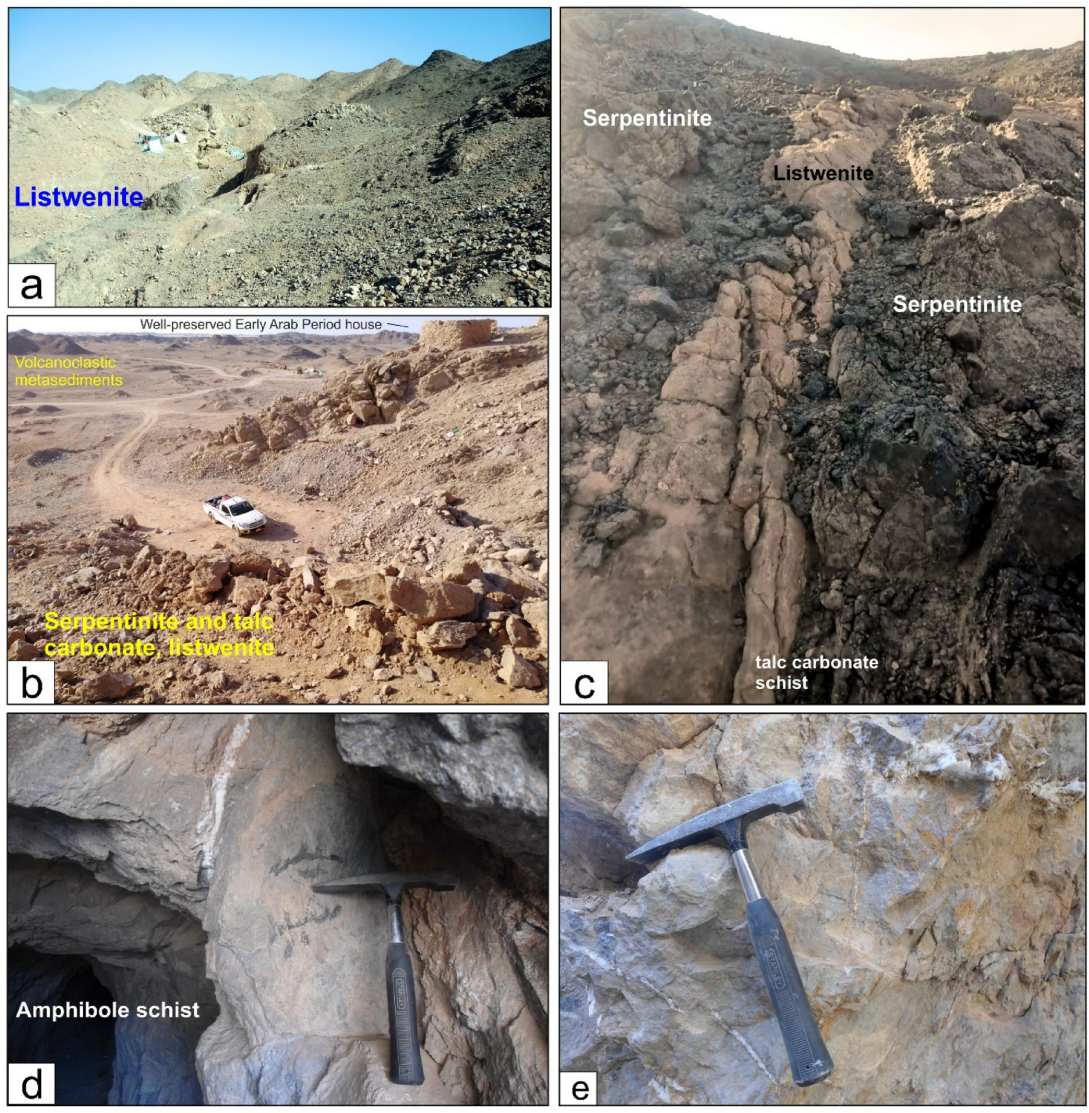
(**a**), (**b**) and (**c**) Serpentinites, listwenite and talc-carbonate schist from the Haimur gold mine area and Wadi El Qulieb, (**d**) Mafic schist, (**e**) Quartz veins in metagabbro.

The ophiolite assemblages predominantly inhabit the western region of the Wadi Allaqi area, manifesting as imbricate thrust sheets and slices (Fig. 1c) comprising serpentinites, talc-carbonate schist (Fig. 2a, b and c), and metagabbros. These slices have been thrust from north to south overlaying the metavolcanics. Notably, the gold deposits are distinctly linked to listwenite rock (Fig. 2a and b) within the western Allaqi shear belt, particularly associated with the NW- and NE-trending allochthonous slices of serpentinite and their corresponding talc-carbonate rocks (Fig. 2b). The serpentinite slices have been thrust over and intermingled with volcanoclastic metasediments and metavolcanics along NE-dipping thrusts (Fig. 2d).

The western Allaqi shear belt is predominantly characterized by volcanoclastic metasediments (Fig. 1c), which include biotite almandine to biotite schists (Fig. 2d), metasiltstone, metamudstone, quartzitic bands^71^, marbles, and lesser amounts of conglomerates, felsic tuffs, and rhyolites. Schists exhibit foliation and significant shearing, often displaying a chevron-like structure. The phyllite in this area is distinguished by its greenish hue, strong foliation, and pencil-like structure. The banding of these rocks is oriented vertically to sub-vertically.

The island arc assemblage is primarily composed of foliated metavolcanics and volcanoclastic metasediments, along with intrusive gabbro to diorite and granodiorite plutons (Fig. 2e). The syn-tectonic granitoids found within the western Allaqi shear belt are interpreted as a result of magmatism associated with tectonic collision^72^. These granitoids are less prevalent than the metavolcanic and volcanoclastic metasediments related to the island arc assemblages. The intrusions exhibit diverse compositions, including tonalite quartz-diorite, diorite, and gabbro (Fig. 2f and g). Additionally, late- to post-tectonic granitic bodies are extensively distributed throughout the central region of the study area, manifesting as deeply eroded, roughly circular formations represented by a few isolated low-lying outcrops of granodiorite (Fig. 2e) that are partially obscured by recent sand deposits.

The most notable marble formations have been located in the western Allaqi shear belt (Fig. 1c), forming conspicuous ridges around the main Haimur anticline. In the neighborhood of Wadi Um Arakah, these marble formations rise to around 25 m in height and span an average width of 200 m. The marbles are found in vertical bands of varying thickness, interspersed with layers of schist^67^. A tectonic contact between schists and marble bands is seen in the eastern portion of Deneibit El Quleib. Two principal variations of marble have been recorded: white marble and gray marble. The white marble has massive strata beyond 20 m, interspersed with schists, and is distinguished by its purity and elevated calcareous content. The black banded marbles, characterized by their graphite content, appear as conspicuous hills with bands that may attain widths up to 5 cm^67^. The metagabbro-diorite (Fig. 1c) is between Wadi Um Ashira and Wadi Haseierbah. Xenoliths are abundant in the contact zones of this metagabbro formation, which is intruded by aplitic and rhyolitic dikes. The granodiorite appears as separate and exfoliated masses, with significant vertical and lateral jointing, which suggests structural deformation. Granodiorite formations occur in intrusive contact with schists along Wadi Umm Ashira. Small gabbroic masses are located on the eastern slope of Wadi Haimur and are characterized by their intrusive contact with the adjacent metavolcanics.

The Haimur gold deposits are located within highly foliated and intensely folded slices of carbonatized ophiolite, which are interspersed among calcareous and carbonaceous volcanoclastic metasediments. The findings from geological mapping and field observations highlight a prominent presence of chert, marble, and magnesite bands correlated with serpentinite, metagabbro, and metabasalt. These geological formations exhibit diverse degrees of silicification and carbonatization in the gold mining sectors^63,73^. Additionally, talc and graphite laminae are frequently found adjacent to the marble bands. Gold-bearing quartz and quartz-carbonate veins are found to be associated with highly tectonized and carbonatized serpentinite and listvenite. The discontinuous quartz lenses stretch more than 25 m along the strike. The mineralization area is characterized by two main quartz veins and host rocks that have undergone significant silicification and carbonatization, resulting in a zone that is roughly 20 m wide^73^.

## Methodology

### Characteristics and analysis of remotely sensed data

#### Data characteristics

The ASTER dataset encompasses a broad spectral range that includes 14 spectral bands. It captures reflected radiation across three bands within the 0.52 to 0.86 μm range (visible-near infrared, VNIR) with a resolution of 15 m, as well as six bands spanning from 1.6 to 2.43 μm (shortwave infrared, SWIR) at a resolution of 30 m. Additionally, emitted radiation is assessed at a resolution of 90 m across five bands within the 8.125 to 11 μm wavelength range (thermal infrared; TIR). The dataset from Landsat-8 OLI includes nine spectral bands, with seven bands focused on measuring reflected radiation in the visible near-infrared (VNIR) and shortwave infrared (SWIR) ranges, each providing a spatial resolution of 30 m for bands 1 through 7 and 9. The panchromatic band 8, in contrast, achieves a resolution of 15 m. The ultra-blue band 1 is highly effective for applications related to coastal areas and aerosols, whereas band 9 plays a crucial role in detecting clouds. Furthermore, the thermal infrared (TIR) bands, specifically bands 10 and 11, capture emitted radiation within the wavelength range of 10.6 to 12.5 µm, offering a spatial resolution of 100 m. The Sentinel-1 satellites are outfitted with C-band Synthetic Aperture Radar (SAR) sensors that function in a dual-polarization mode, encompassing both co-polarized (VV or HH) and cross-polarized (VH or HV) configurations. These sensors operate in an interferometric wide-swath (IW) mode, achieving a spatial resolution of 5 × 20 m.

The study area was analyzed using freely available cloud-free satellite imagery, which includes Landsat-8 L1TP data collected on February 27, 2025 (path 174/row 44), four ASTER L1T scenes from August 4, 2000, Sentinel-1B (S1B) Level-1 Ground Range Detected (GRD) imagery acquired on September 14, 2021, and one Digital Elevation Model (DEM) scene obtained on February 2, 2000. The optical datasets, comprising Landsat-8 OLI and ASTER imagery, were utilized for lithological mapping, identification of alteration zones, and structural analysis. In contrast, the S1B radar data facilitated the automatic extraction of lineaments. Additionally, the DEM data contributed to creating a hillshade map for the study area.

#### The preliminary processing of satellite data

The optical bands from Landsat-8, bands explicitly 2 to 7, in conjunction with ASTER bands 1 to 9, as well as S1B radar and Digital Elevation Model (DEM) datasets, were subjected to layer stacking, mosaicking, subsetting, and processing through ENVI 5.3, ArcMap, PCI Geomatica, and RockWork software. This comprehensive approach aimed to highlight the study area and enhance lithological discrimination, alteration mapping, and lineament extraction. The optical, radar, and DEM datasets were geometrically reprojected to the Universal Transverse Mercator (UTM) projection, specifically Zone N36, utilizing the WGS-84 datum. Cross-track illumination was employed for the Landsat-8 OLI data to address atmospheric effects. At the same time, the Internal Average Relative Reflection (IARR) algorithm was utilized for ASTER data to reduce atmospheric impacts and convert radiance data into surface reflectance. Additionally, the Enhanced Lee filter was applied to the S1B radar data within ENVI to reduce speckle noise, and the DEM data underwent pre-processing in ArcMap 10.8 using the Filling order feature within the spatial analysis tools.

#### The analysis of satellite data

A wide range of image processing techniques was implemented to enhance the visibility of lithological interfaces among different rock units and extract critical structural features in the examined area. These included False Color Composite (FCC), Decorrelation Stretch (DS), Principal Component Analysis (PCA), and Minimum Noise Fraction (MNF), which were applied to optical satellite data to create an exact geological map. Additionally, various band ratios from Landsat-8, precisely 6/7, 7/5, 6/4, and 6/5, along with the logarithmic Constrained Energy Minimization (CEM) technique applied to ASTER data, were employed to identify alteration zones, thereby revealing specific locations of gold deposits.

The study uses Principal Component Analysis (PCA) and Minimum Noise Fraction (MNF) techniques to identify different lithological units and geological formations in multispectral imagery. PCA reveals uncorrelated linear combinations of variables^74^, while MNF reduces noise and isolates it within the dataset^75^. Decorrelation Stretch enhances color differentiation and mitigates correlation among bands. Band Ratios (BR) highlights specific areas of interest by dividing two corresponding spectral bands with spatial resolution. This study successfully used BR on Landsat-8 data to identify hydrothermal alteration minerals. The integration of the CEM technique with the ENVI spectral tool allowed for the detection of alteration minerals and their spatial distribution within a defined area, based on the diagnostic spectral characteristics of each mineral.

The processing of S1B radar data involved Band Math (BM), which resulted in the fusion of VV and VH polarizations into a composite layer (VV + VH). This composite layer was subsequently combined with the individual polarizations (VV and VH) to form a stacked configuration. Following this, Principal Component Analysis (PCA) was performed on the stacked layers (VV, VH, and VV + VH), leading to the derivation of three principal components (PC1, PC2, and PC3). The first principal component (PC1) was then subjected to analysis using the LINE algorithm function within PCI Geomatica software, which facilitated the automated extraction of structural elements, specifically lineaments, guided by a gradient threshold parameter of 50.

### Magnetic

EDs of potential field methods can provide reliable interpretation and delineation of subsurface structures^20,31,76,77^. Cooper and Cowan^78^ introduced the horizontal tilt angle (TDX) for sharp edge detection. Ibraheem et al.^79^ introduced an improved version of TDX (Im-TDX) and computed the total-horizontal derivative (HTDR) of Im-TDX (HTDR_Im-TDX).

1 $$TDX = tan^{ - 1} \left( {\frac{{\sqrt {\left( {\frac{\partial f}{{\partial x}}} \right)^{2} + \left( {\frac{\partial f}{{\partial y}}} \right)^{2} } }}{{\left| {\frac{\partial f}{{\partial z}}} \right|}}} \right)$$

where ${{\partial f} \mathord{\left/ {\vphantom {{\partial f} {\partial x}}} \right. \kern-0pt} {\partial x}}$, ${{\partial f} \mathord{\left/ {\vphantom {{\partial f} {\partial y}}} \right. \kern-0pt} {\partial y}}$, and ${{\partial f} \mathord{\left/ {\vphantom {{\partial f} {\partial z}}} \right. \kern-0pt} {\partial z}}$ are field derivatives in *x, y,* and *z* directions.

2 $$Im - TDX = tanh\left( {\frac{{ - M.\left( {\frac{{\partial^{2} f}}{{\partial x^{2} }} + \frac{{\partial^{2} f}}{{\partial y^{2} }}} \right)}}{{\sqrt {\left( {\frac{\partial TDX}{{\partial x}}} \right)^{2} + \frac{\partial TDX}{{\partial y}}^{2} } }}} \right)$$

where *M* is the average intensity value of magnetic field.

3 $$THDR\_Im - TDX = \sqrt {\left( {\frac{\partial Im - TDX}{{\partial x}}} \right)^{2} + \left( {\frac{\partial Im - TDX}{{\partial y}}} \right)^{2} }$$

Pham^45^ introduced a stable filter to detect edges depending on the first-order derivatives’ hyperbolic-tangent function (HTF).

4 $$HTF = tanh\frac{{ - 2 \left| {\frac{\partial f}{{\partial z}}} \right|}}{{\sqrt {\left( {\frac{\partial f}{{\partial x}}} \right)^{2} + \left( {\frac{\partial f}{{\partial y}}} \right)^{2} } }}$$

Pham^46^ suggested a novel edge detector (NED) depending on derivatives of horizontal gradient (HG)^80^.

5 $$NED = \frac{1}{2}\left( {{\text{atan}}\frac{{\frac{\partial HG}{{\partial z}} - \sqrt {\left( {\frac{\partial HG}{{\partial x}}} \right)^{2} + \left( {\frac{\partial HG}{{\partial y}}} \right)^{2} } }}{{\sqrt {\left( {\frac{\partial HG}{{\partial x}}} \right)^{2} + \left( {\frac{\partial HG}{{\partial y}}} \right)^{2} } }} + {\text{atan}}\frac{{\sqrt {\left( {\frac{\partial HG}{{\partial x}}} \right)^{2} + \left( {\frac{\partial HG}{{\partial y}}} \right)^{2} + \left( {\frac{\partial HG}{{\partial z}}} \right)^{2} } }}{{\sqrt {\left( {\frac{\partial HG}{{\partial x}}} \right)^{2} + \left( {\frac{\partial HG}{{\partial y}}} \right)^{2} } }}} \right)$$

where, HG = $$\sqrt {\left( {{{\partial f} \mathord{\left/ {\vphantom {{\partial f} {\partial x}}} \right. \kern-0pt} {\partial x}}} \right)^{2} + \left( {{{\partial f} \mathord{\left/ {\vphantom {{\partial f} {\partial y}}} \right. \kern-0pt} {\partial y}}} \right)^{2} }$$.

**Fig. 3:**
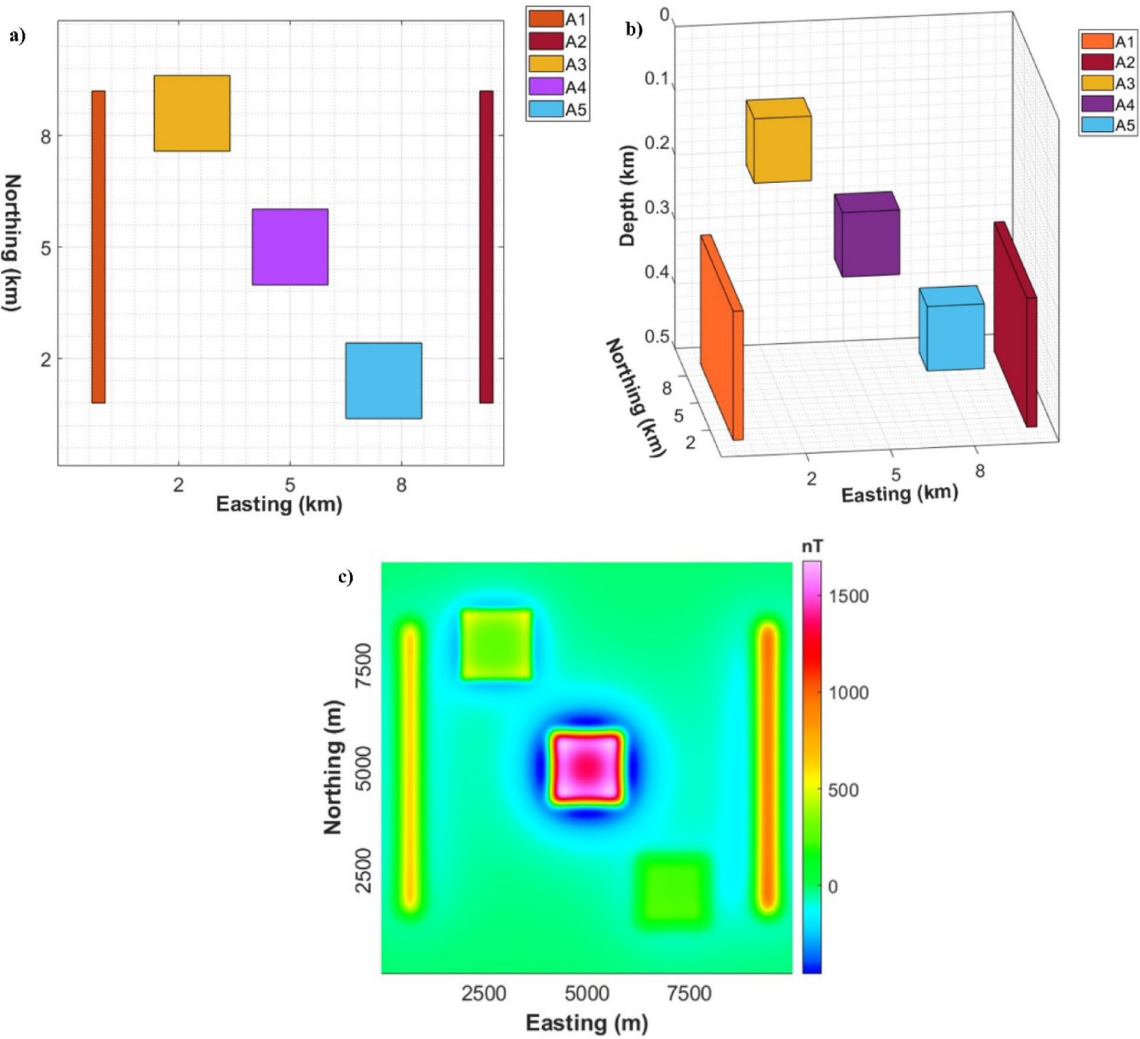
(**a**) Synthetic model, (**b**) 3D view of model, and (**c**) Magnetic anomaly of the simulated model.

To evaluate their robustness and accuracy in mapping deep structures, the THDR_Im-TDX, Im-TDX, HTF, and NED filters will be applied to a synthetic model as shown in Fig. 3 and Table 1. Then, we will use the most sharp and accurate ones for the reduced-to-pole (RTP) data of the western Allaqi shear belt area^81^.

**Table 1:** Parameters of simulated models.

Parameters	A1	A2	A3	A4	A5
Mag susceptibility	0.2000	− 0.3000	0.1000	− 0.5000	− 0.1000
Prism width (m)	300	300	1700	1700	1700
Prism length (m)	7000	7000	1700	1700	1700
Prism thickness (m)	200	200	100	100	100
X coordinates (m)	700	9400	2800	5000	7100
Y coordinates (m)	5000	5000	8000	5000	2000
Depth to top (m)	300	300	100	200	300
Rem. Inc & decli (deg)	90, 0	90, 0	90, 0	90, 0	90, 0

## Results

### Remotely sensed data

#### Delineation of lithological contacts via Landsat-8

The use of colored images in lithological classification has been enhanced by composites such as Decorrelated FCC 657, MNF 324, and PCs 132 and 342 in RGB. These composites help identify key structural elements and improve existing geological maps for the region under investigation, enhancing the visual differentiation of lithological features and structural attributes.

**Fig. 4:**
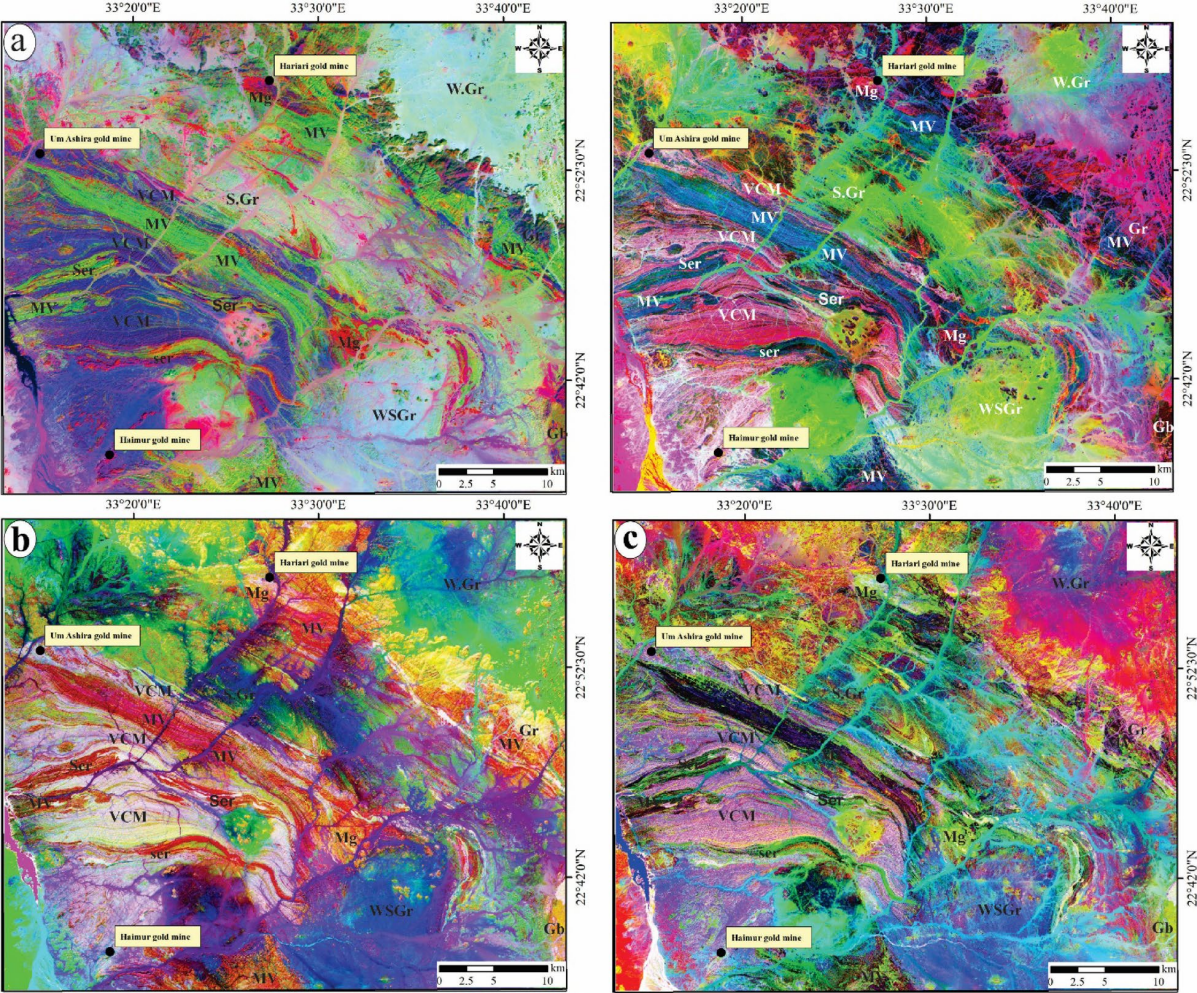
Lithological delineation by Landsat-8, (**a**) Decorrelated FCC 657; (**b**) MNF 324; (**c**) PC 132; and (**d**) PC 342 in RGB. Abbreviation: Ser = serpentinite and talc carbonates, MV = felsic to intermediate metavolcanics, VCM = volcanoclastic metasediments, Mg = syn-tectonic metagabbro, WSGr = weathered syn-tectonic granite, SGr = syn-tectonic granite, WGr = weathered post-tectonic granite, Gr = post-tectonic granite, Gb = post-tectonic gabbro.

.The decorrelated FCC 657-RGB (Fig. 4a) effectively delineates the lithological boundaries among various rock units, with serpentinite and talc carbonates represented in shades of orange to pink, volcanoclastic metasediments depicted in dark blue, metavolcanics illustrated in green to yellowish green, syn-metagabbro shown in a bloody red hue, post-gabbro in orange-green, syn-granite in greenish white, weathered syn-granite in cyan, post-granite in violet, and weathered post-granite in bluish white. Furthermore, the MNF 343-RGB (Fig. 4b) clearly reveals the distinct contacts between the ophiolitic sequence, characterized by dark brown tones of serpentinites and talc carbonates, and the island arc assemblages, which include arc metavolcanics in blue and volcanoclastic metasediments in pink. The syn- and post-magmatic sequences are differentiated by a palette of reddish violet, bluish white, green, pinkish violet, cyan, and reddish brown, corresponding to syn-metagabbro, syn-granite, weathered syn-granite, post-granite, weathered post-granite, and post-gabbro, respectively (Fig. 4b).

Figures 4c and d illustrate the lithological boundaries between the ophiolitic sequence, arc assemblages, and syn-post magmatic sequence in both Principal Component (PC) 132 and PC 342 using RGB color modes. In the PC 132-RGB representation (Fig. 4c), the lithological units are identified with specific colors: yellowish green with red bands for the ophiolitic sequence, reddish pink for the arc metavolcanics, pink for the volcanoclastic metasediments, orange for the syn-metagabbro, brass yellow for the post-gabbro, yellowish green for the syn-granite, deep bluish violet for weathered syn-granite, shiny yellow for post-granite, and violet-cyan for weathered post-granite. Conversely, in the PC 342-RGB depiction (Fig. 4d), the arc metavolcanics are notably more prominent, represented by a deep violet hue, contrasting with the light violet color of the volcanoclastic metasediments. Additionally, the weathered syn-granitoids are distinctly marked by a blue color, while a pinkish blue shade characterizes the weathered post-granitoids.

#### Remotely sensed data inform the distribution of alteration zones.

The hydrothermal mineral assemblages present in these altered zones, which include clay, carbonate, iron oxides, and minerals rich in Fe and Mg-OH, are primarily associated with altered basic-ultrabasic rocks and display distinct spectral signatures. Conversely, the Al–OH mineral groups, such as clay, alunite, and muscovite, are found within the altered felsic rocks. The recognition of OH-hydrous minerals (Mg-OH, Al–OH, and Si–OH) along with the CO3 group is enhanced by their unique absorption characteristics in the shortwave infrared (SWIR) spectrum, specifically within the range of 2.0 to 2.50 micrometers^82–84^.

(i) Allocation of alteration zones via Landsat-8

**Fig. 5:**
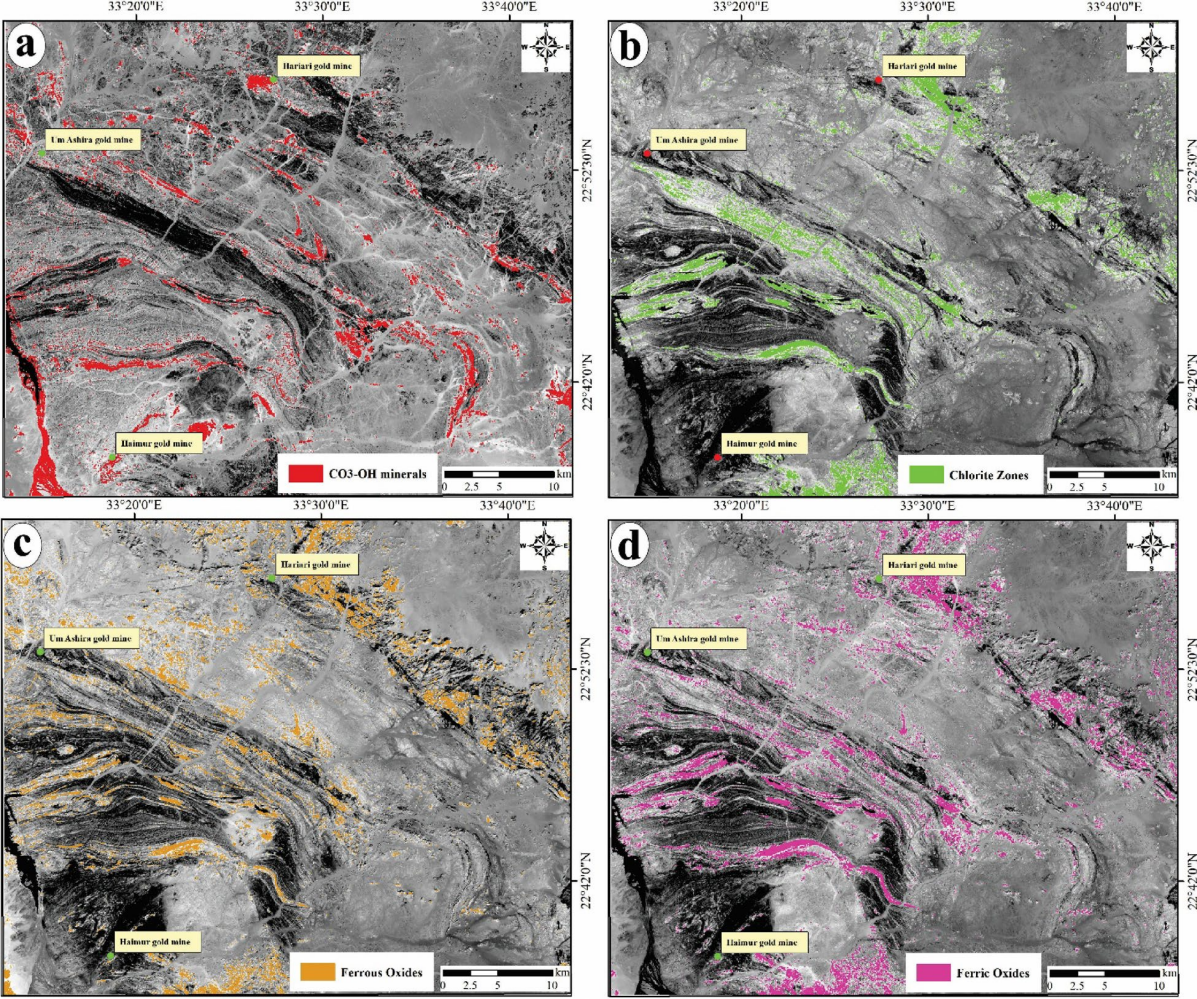
Grey-scale BRs of Landsat-8, (**a**) 6/7 for hydroxyl-bearing alterations; (**b**) 7/5 (representing the chlorite zones; (**c**) 6/4 indicative of ferrous iron oxides and (**d**) 6/5 representing ferric oxides.

A variety of band ratios, specifically 6/7, 7/5, 6/4, and 6/5, were utilized in grey scale mode (Fig. 5a, b, c and d) to delineate the general alteration zones and their spatial distributions across the various rock units within the study area. These alteration zones are represented by bright pixels, which were subsequently enhanced with colored patches using ENVI software for improved visibility. The band ratio 6/7 was specifically employed to identify regions enriched with CO3 and OH-bearing minerals (Fig. 5a). In contrast, the band ratio 7/5 was used to emphasize the chlorite-rich areas (Fig. 5b). Additionally, the regions characterized by ferric and ferrous iron oxides were identified using band ratios 6/4 and 6/5, respectively. All identified zones containing CO3 and OH-bearing minerals, chlorite, and iron oxides are notably associated with ore deposits, including gold deposits. It is noticed from the BRs that most of the altered areas were occupied by the southwestern sector of the study areas, which are dominated by serpentinites, talc carbonates, felsic to intermediate metavolcanics, volcanoclastic metasediments, and syn-metagabbro, as well as some parts along the northeastern parts of felsic to intermediate metavolcanics composition.

(ii) Allocation of alteration zones via ASTER

**Fig. 6:**
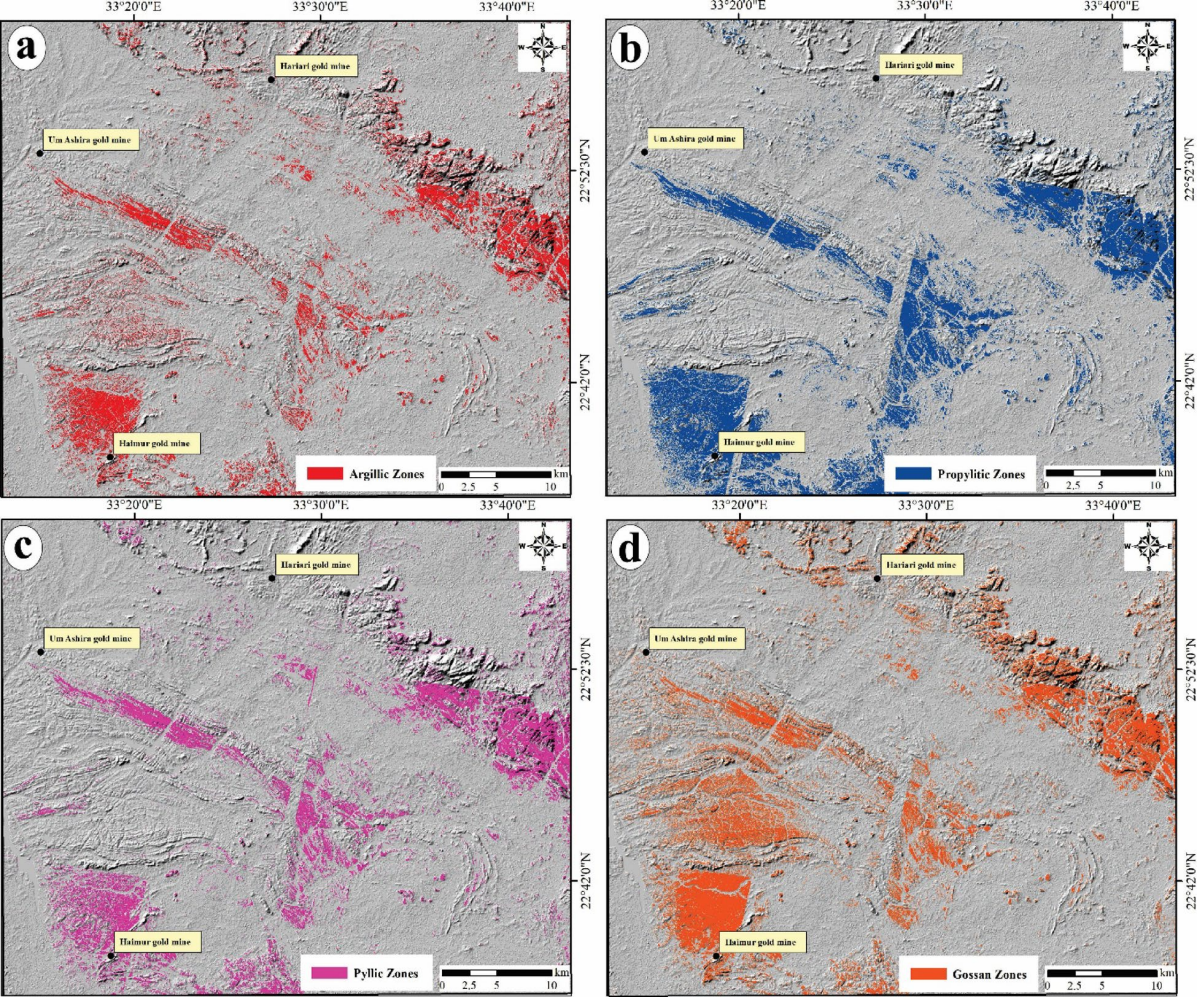
Spatial distribution of the four standard alteration zones via CEM using ASTER data, (**a**) Argillic zones; (**b**) Phyllic zones; (**c**) Propylitic zones and (**d**) Gossan zones.

The Constrained Energy Minimization (CEM) technique has been applied on the first nine VNIR + SWIR bands of the ASTER data to allocate the surface distribution for thirteen of the alteration minerals which are marked the four standard alteration zones enclosing i) the argillic zone (kaolinite, illite, alunite and, montmorillonite); ii) phyllic zone (illite, muscovite and biotite); iii) propylitic zone (chlorite, epidote, calcite and talc) and iv) gossan zone i (hematite, goethite and jarosite). By adjusting the rule threshold (Table 2) through the CEM method and utilizing the spectral library provided by the USGS, grey-scale images were generated to emphasize the surface distribution of thirteen alteration minerals. These minerals were subsequently aggregated and represented as four distinct alteration zones, depicted as color patches across the specified area (see Fig. 6a, b, c and d), enhancing their respective locations’ clarity. It is noticed that the spatial distribution of the four alteration zones was recorded in the southwestern and northeastern sectors of the investigated area which are characterized by ophiolitic, arc assemblages and syn-tectonic metagabbro.

**Table 2:** Statistic table displays a brief summary of the alteration minerals using the CEM technique in the study area.

Alteration zone	Mineral	Method	Rule Threshold	Target Count	Average area km^2^
Argillic zone	Kaolinite	CEM	0.090	16,741	7308.1655
	Illite		0.090	10,639	5889.6958
	Alunite		0.180	25,647	4459.7319
	Montmorillonite		0.090	13,640	5879.1479
Phyllic zone	Illite		0.090	10,639	5889.6958
	Muscovite		0.090	18,904	8031.5381
	Biotite		0.090	17,808	8612.3018
Propylitic zone	Calcite		0.090	11,620	7101.8481
	Talc		0.090	11,406	6537.6250
	Chlorite		0.090	19,171	11407.198
	Epidote		0.090	16,326	15399.173
Gossan zone	Goethite		0.220	28,012	7424.4697
	Hematite		0.220	27,422	7829.4043
	Jarosite		0.090	15,895	3.1

#### S1B based-lineaments extraction

**Fig. 7:**
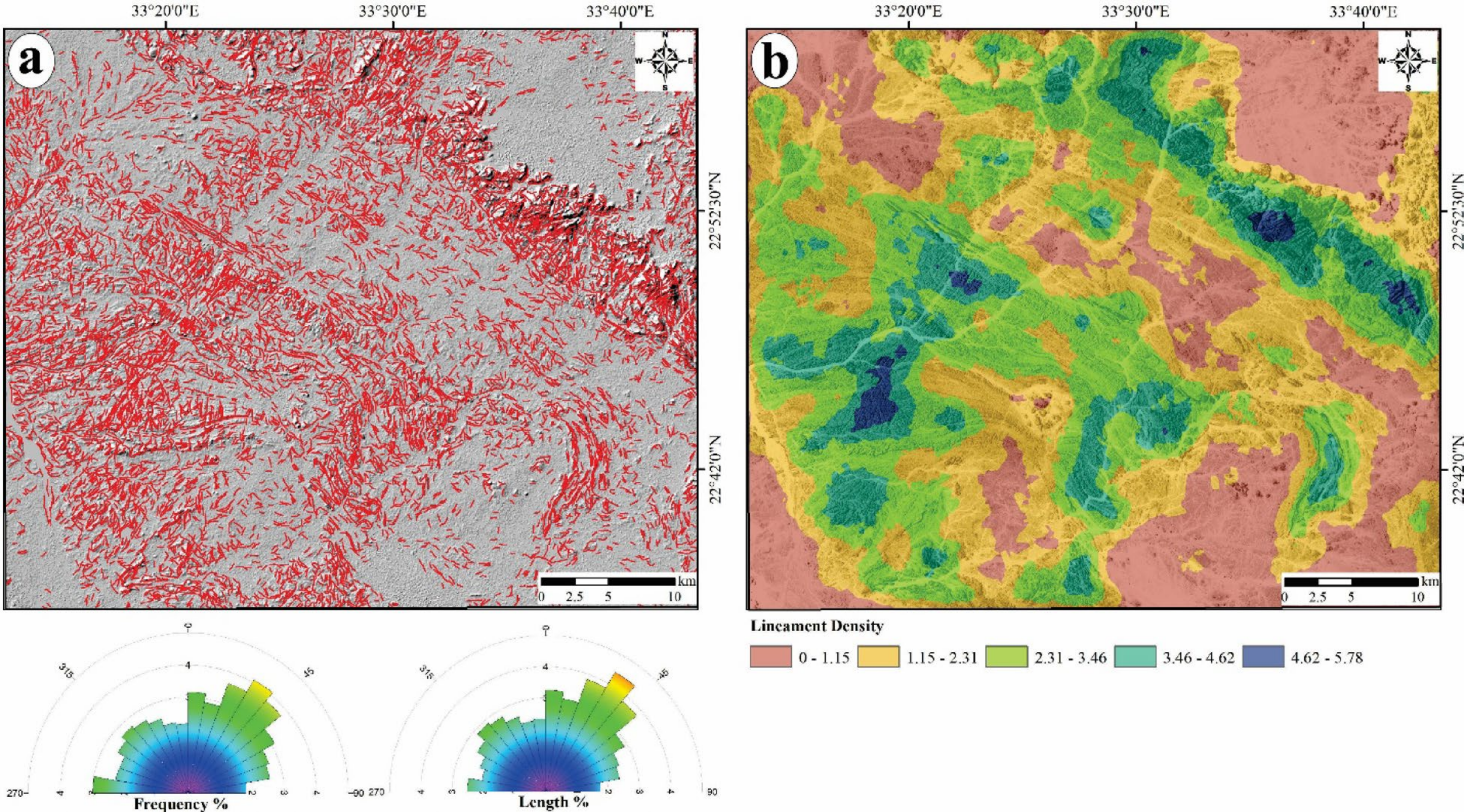
Automatic lineaments extraction, (**a**) Surface distribution of lineaments dropped over a Hillshaded map with frequency and length azimuth diagram, and (**b**) Density map for the extracted lineaments.

The analysis of S1B radar data has yielded principal component (PC1) that has been utilized for the automated extraction of lineaments (Fig. 7a). This approach has facilitated the development of a lineament map that illustrates the surface distribution of various geological features, such as faults, joints, and dykes, represented as short, dense red lines. Additionally, azimuth rose diagrams have been produced using Rockwork software for the specified region (Fig. 7a). A lineament density map has also been constructed, based on the spatial distribution of the extracted lineaments, which highlights the concentrations of these geological features (Fig. 7b). The azimuth frequency and length diagrams for the area under investigation reveal that the dominant trends of the lineaments/fractures are primarily oriented in the following decreasing order: NE, NNE, N-S, WNW, and NW.

### Aeromagnetic analysis

**Fig. 8:**
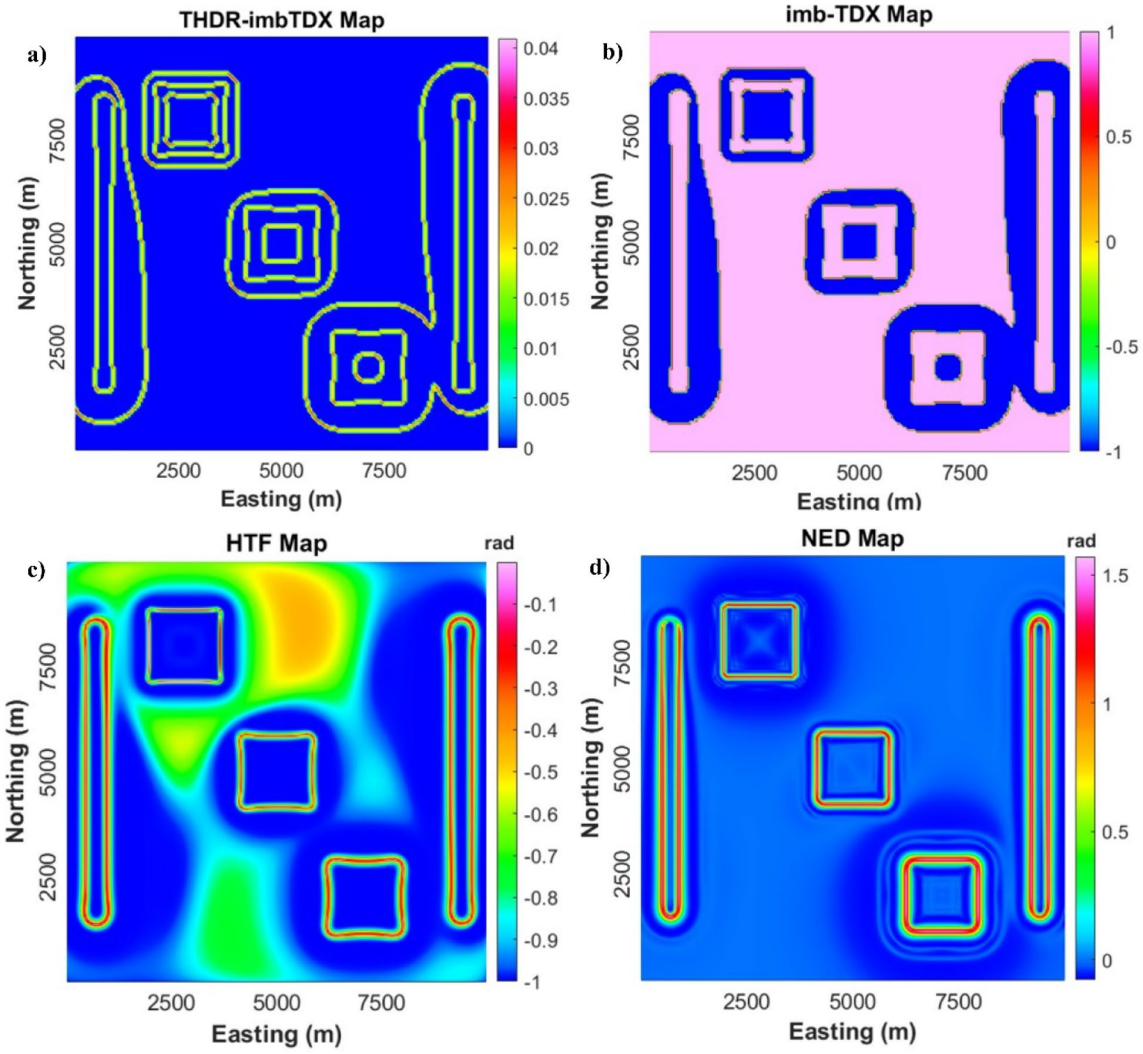
Edges of synthetic anomalies in Fig. 1: (**a**) THDR_Im-TDX; (**b**) Im-TDX; (**c**) HTF; and (**d**) NED.

In our study, multiple ED approaches, THDR_Im-TDX, Im-TDX, HTF, and NED, were tested on synthetic magnetic examples (Fig. 8). We performed these filters to assess their robustness in structural delineation. The results demonstrate that while the THDR_Im-TDX and Im-TDX filters (Fig. 8a and b) produce sharp boundaries, they also generate multiple false boundaries, leading to tricky structural understandings. In contrast, the HTF and NED methods (Fig. 8c and d) reveal superior precision by outlining the source’s edges without any false detections.

**Fig. 9:**
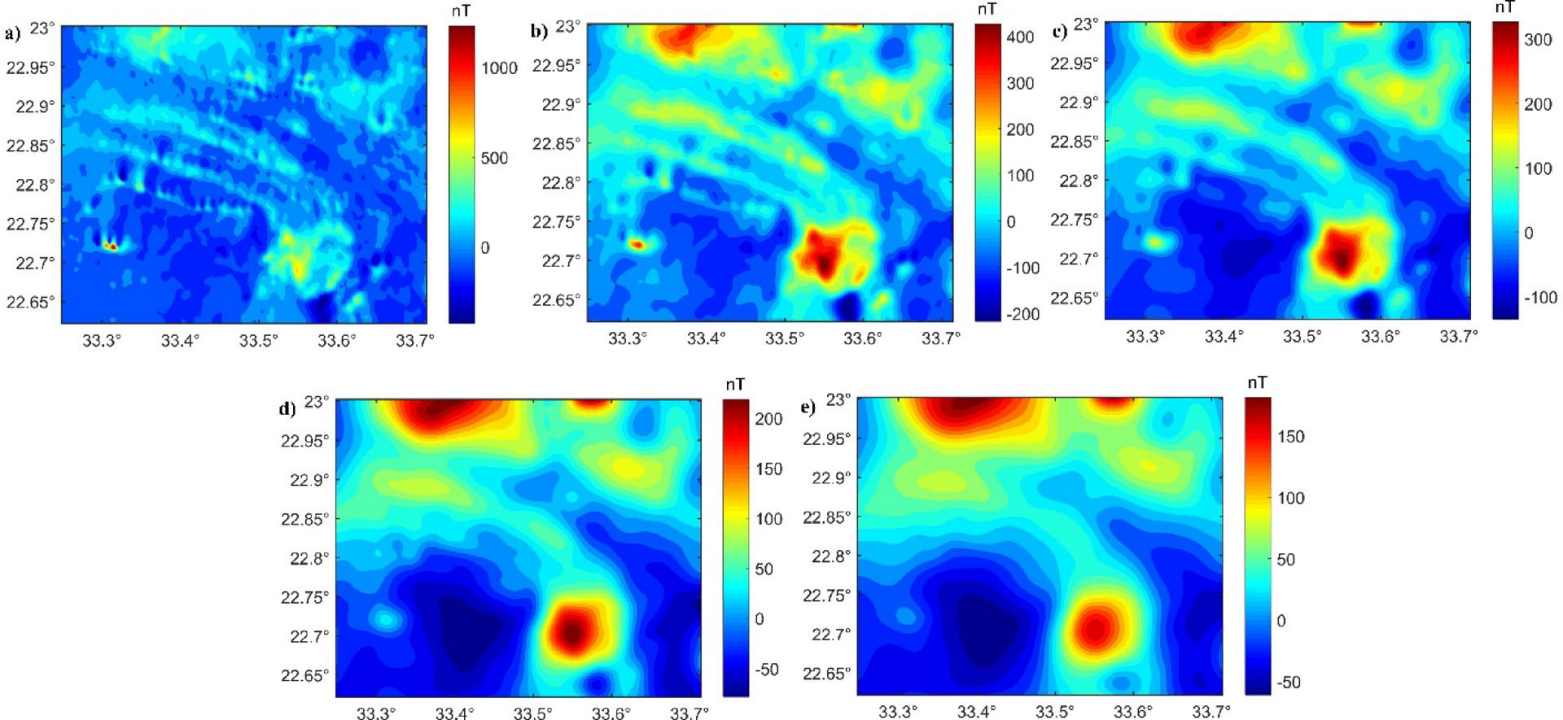
(**a**) RTP; (**b**) UWC-RTP at 0.5; (**c**) UWC-RTP at 1 km; (**d**) UWC-RTP at 1.5 km; and (**e**) UWC-RTP at 3 km of the Western Allaqi shear belt.

**Fig. 10:**
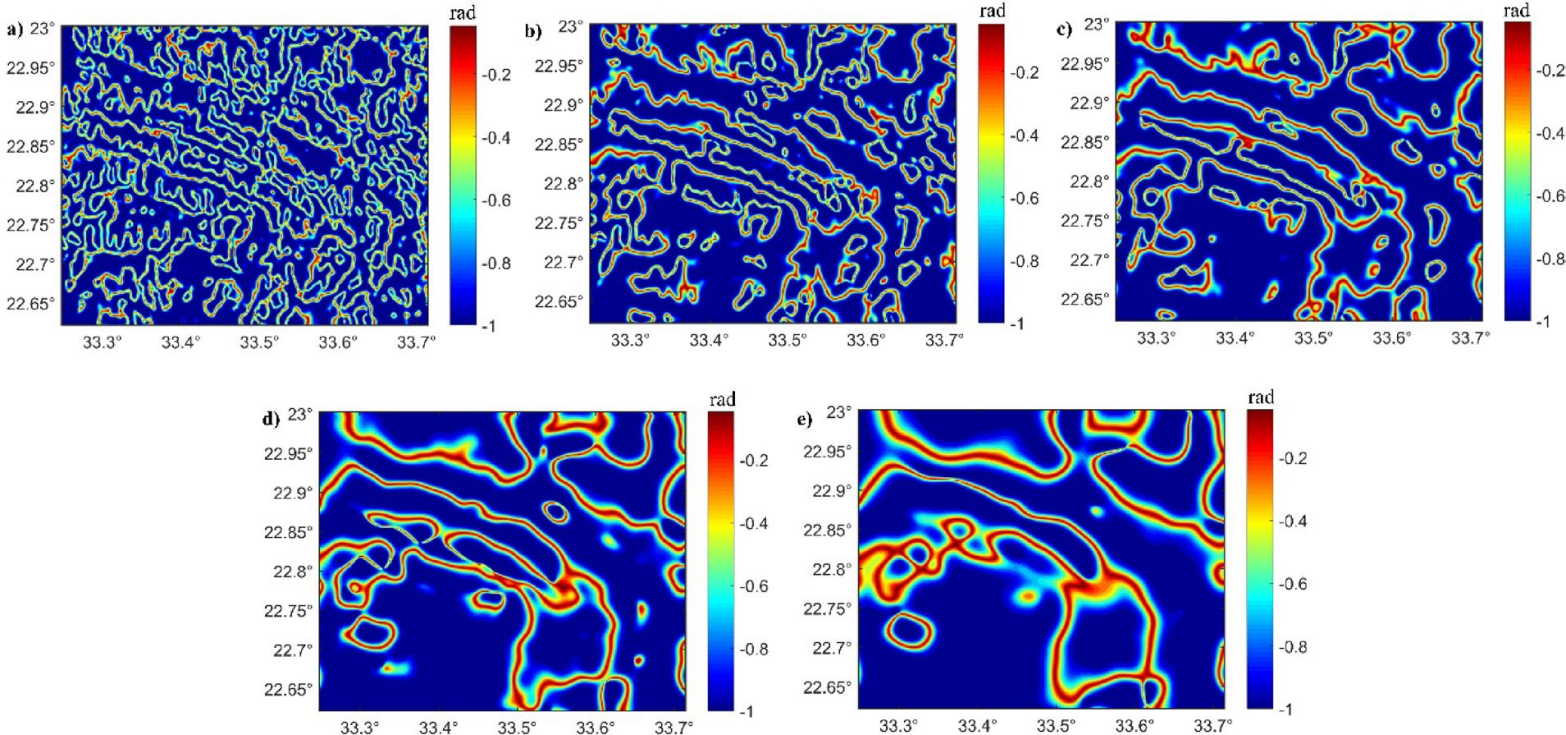
(**a**) HTF of RTP; (**b**) HTF of UWC-RTP at 0.5; (**c**) HTF of UWC-RTP at 1 km; (**d**) HTF of UWC-RTP at 1.5 km; and (**e**) HTF of UWC-RTP at 3 km of Western Allaqi shear belt.

**Fig. 11:**
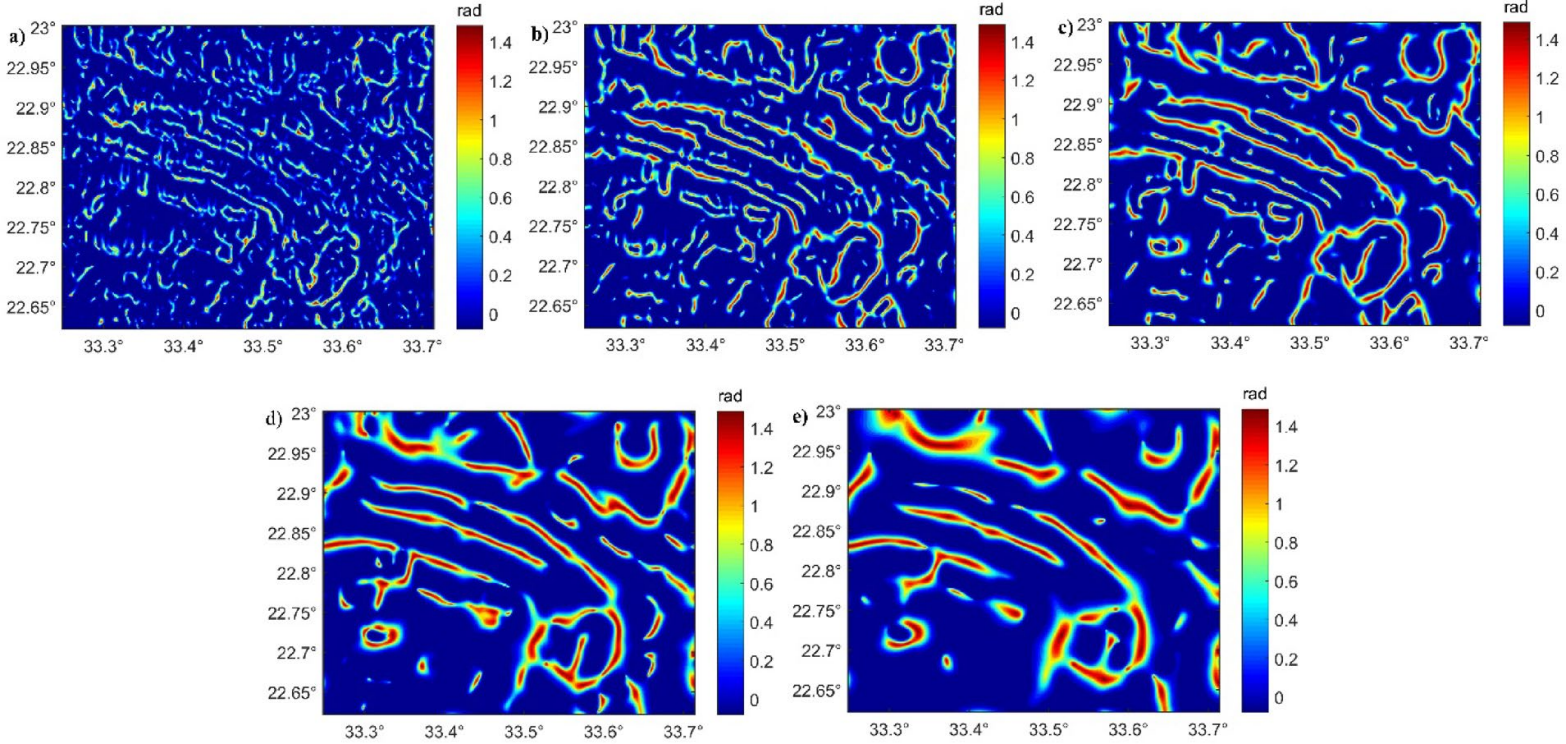
(**a**) NED of RTP; (**b**) NED of UWC-RTP at 0.5; (**c**) NED of UWC-RTP at 1 km; (**d**) NED of UWC-RTP at 2 km; and (**e**) NED of UWC-RTP at 3 km of Western Allaqi shear belt.

To delineate the deep-seated and shallow structures of western Allaqi shear belt, the RTP data (Fig. 9a) are upward-continued (UWC) to depth of 0.5, 1, 1.5, and 3 km (Fig. 9b–e)^26,40,85–88^. Therefore, the HTF and NED filters were applied to real RTP- and UPC-RTP data (Figs. 10 and 11) to provide a reliable and precise understanding of the structural setting controlling gold deposits.

**Fig. 12:**
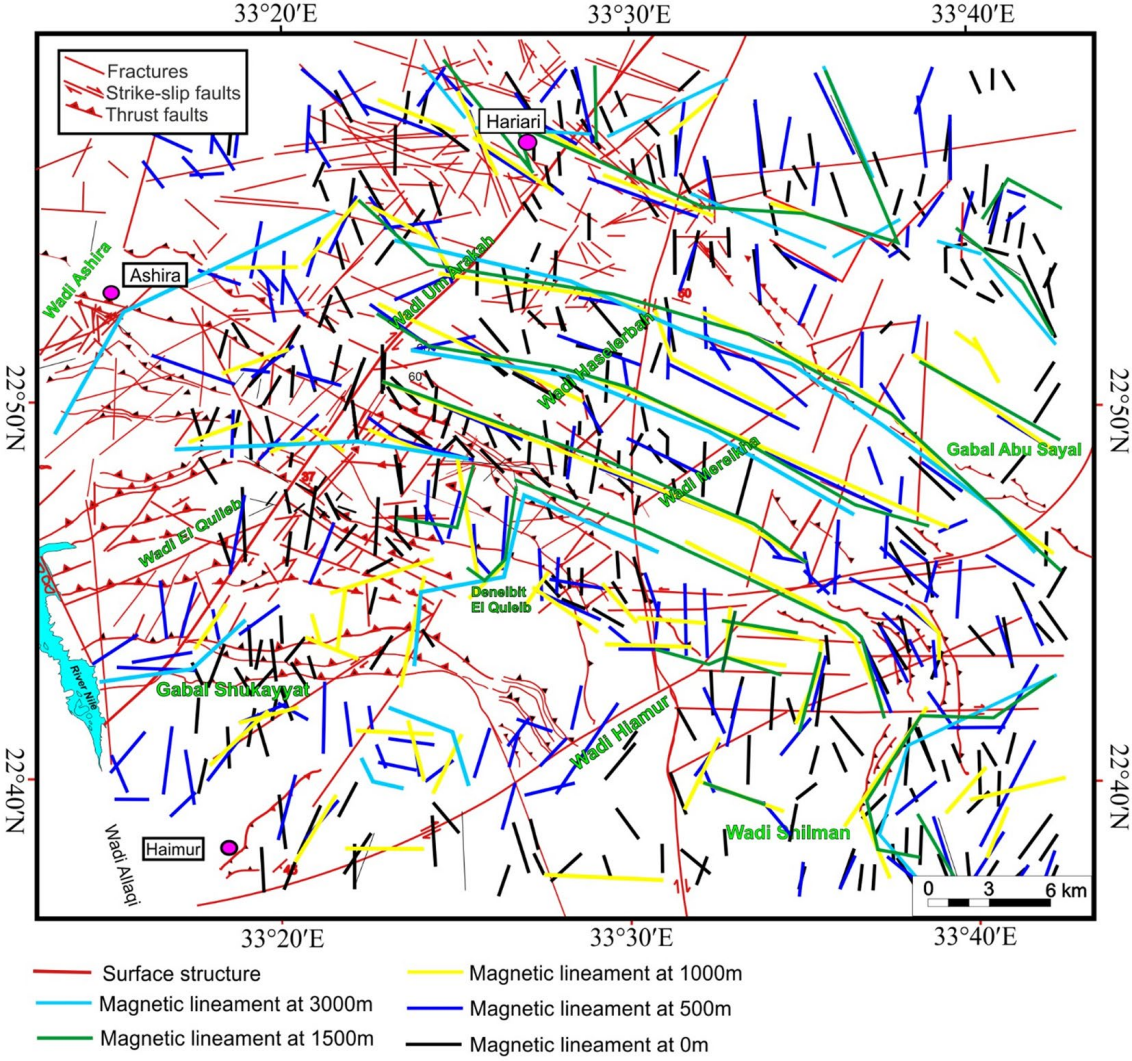
Traced structures and lineaments from Figs. 10 and 11.

**Fig. 13:**
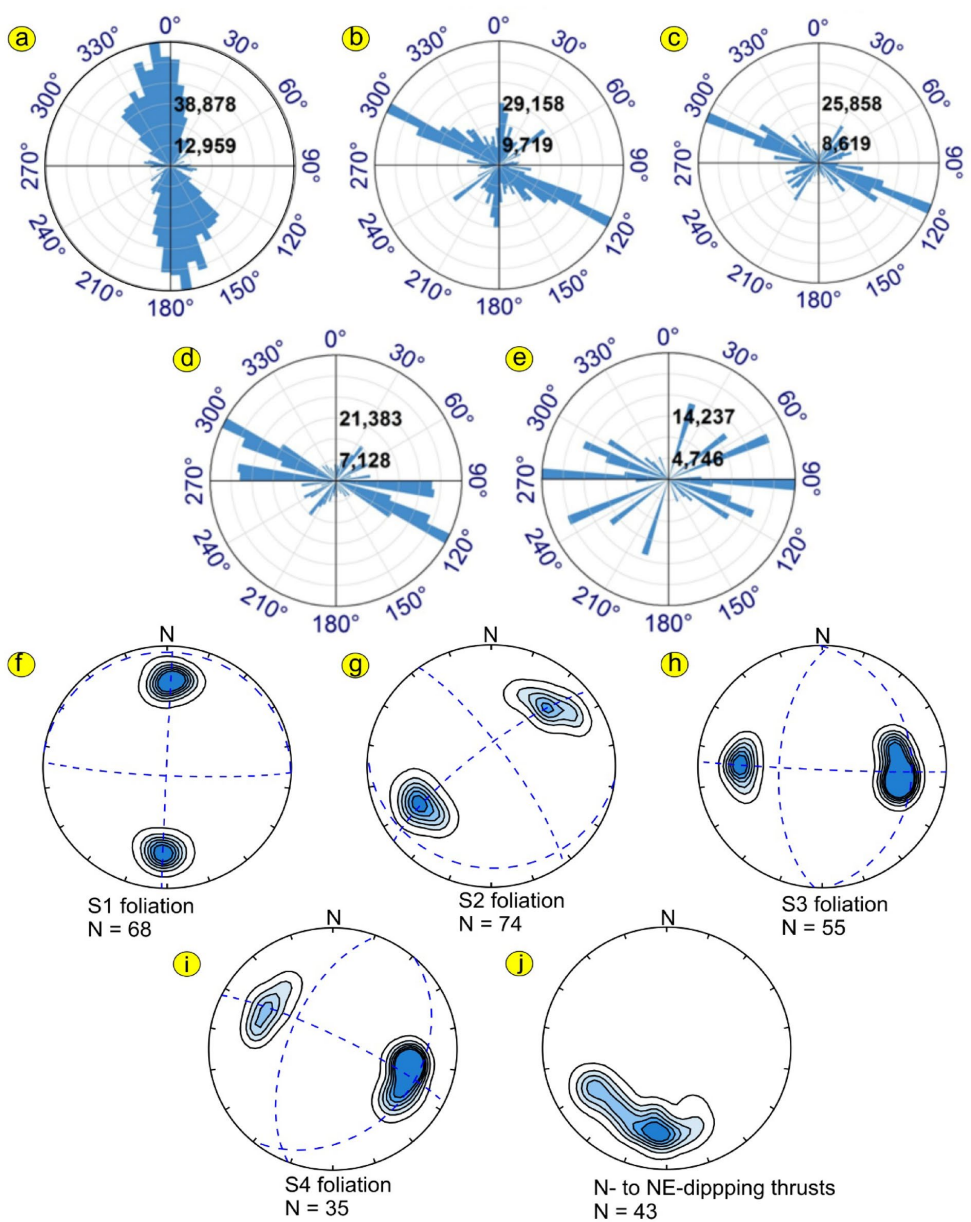
Rose diagrams of lineaments in Fig. 6: (**a**) HTF and NED of RTP; (**b**) HTF and NED of UPW at 0.5 km; (**c**) HTF and NED of UWC at 1 km; (**d**) HTF and NED of UWC at 1.5 km; and (**e**) HTF and NED of UWC at 3 km. Planar structural data collected from the western Allaqi shear belt. Data is plotted stereographically projected onto the lower hemisphere of a Schmidt net. Dashed great circles are the best-fit girdles, and the red star symbols represent the girdle axes, contour intervals: 0, 5, 10, 15, 20, 25. Diagrams (**f**) Poles to S1 regional foliation, (**g**) Poles to S2 foliation, (**h**) Poles to S3 foliation, (**i**) Poles to S4 foliation, and (**j**) Poles to N- and NE-dipping thrusts.

The traced lineaments from Figs. 10 and 11 are shown in Fig. 12 and statistically presented as rose diagrams (Fig. 13). The lineaments and structures (Figs. 10, 11, 12, and 13) showed that the N-S to NW directions are the main trends dominating the area. At depths of 0.5, 1, and 1.5 km, the dominant direction is the WNW trend with N-S, NE, NW, and E-W directions. At a depth of 3 km, the WNW, E-W, ENE, NE, NNE, and NW are the prevailing structures of the western Allaqi shear belt area. Moreover, the WNW prevails in the west and central parts, while the N-S dominates on the area’s eastern side. The HTF and NED clearly outlined the strike-slip faults, folds, and thrusts that control the contacts between various rock units compared to those in the geological map (Fig. 1c).

## Structural framework of the western Allaqi shear belt

Abd El-Wahed^89^ has structurally classified the Egyptian Nubian Shield into three primary domains (Fig. 1a): the Northern Extensional Domain (NED), the Central Transpressional Domain (CTD), and the Southern Compressional Domain (SCD). The Allaqi shear belt serves as the main feature of the SCD. The Neoproterozoic deformation within the western Allaqi shear belt has garnered considerable attention in the academic literature^67,71,90–96^. El Kazzaz^91^ identified five distinct phases of deformation in the Wadi Allaqi region, designated as D1 through D5. The initial stages, D1 and D2, represent the main tectonic events, whereas D3 through D5 indicate subsequent deformation processes. The D1 phase is marked by NW-trending features, with SE-oriented shear zones display penetrative foliation and lineation, plunging approximately to the northeast. In contrast, D2 is characterized by east–west shear trends. The D3 phase is identified by NE-SW-trending folds that include kink bands. D4 is noted for its minor structural characteristics and conjugate fractures, which generally trend NW–SE. Lastly, the D5 phase consists of a series of sinistral strike-slip faults trending north-northeast to south-southwest, which displace the earlier dextral strike-slip faults trending east–west. Abdel-Meguid et al.^93^ identified four separate nappe complexes, classifying them into the upper complex, which features serpentinite and metapyroxenites; the middle complex, mainly consisting of sillimanite schist, metavolcanics, and metaproclastics; and the southern complex, which is predominantly made up of mafic gneisses and quartzofeldspathic mylonites. In a subsequent study, Abdelsalam et al.^96^ proposed a tripartite deformation model within the western Allaqi shear belt. The initial two deformation events are interpreted as a singular continuous process, with a later folding phase occurring atop the earlier thrusting. A general east–west shortening characterizes the third event. The sequence of deformation is outlined as follows: (1) the emplacement of nappes from the north or northeast toward the south or southwest; (2) the refolding of the central allochthon around subhorizontal, northwest-trending axes; and (3) the refolding of the southern allochthon around north-trending axes.

Abd El-Wahed et al.,^16^ identified four generations of foliation in the WASB. S1 displays an approximate east–west orientation (Fig. 13f), which is succeeded by foliation trends of NW–SE (S2, Fig. 13g), N-S (S3, Fig. 13h), and NE-SW (S4, Fig. 13i). By integrating our field observations with magnetic and remote sensing data, we can outline the structural evolution of the region into four main deformational stages following Abd El-Wahed et al.^16^. These stages are (1) a nearly N-S shortening phase (D1)^16^, characterized by N- to NE-dipping thrusts (Fig. 13j) and the development of approximately E-W mineral foliations and folds within the ophiolitic and volcanoclastic metasediments^16^; (2) an E-W shortening phase (D2-D4) that transitioned into a simple shear phase, resulting in kilometer-scale folds and NW-trending sinistral shear zones^16^, alongside N-S and NE-trending dextral shear zones, thereby forming a three-component system within a prominent NW-strike-slip sinistral shear system, likely linked to the Najd Fault System; and (3) the development of brittle strike-slip faults (D5)^16^ that were superimposed on pre-existing discontinuities.

**Fig. 14:**
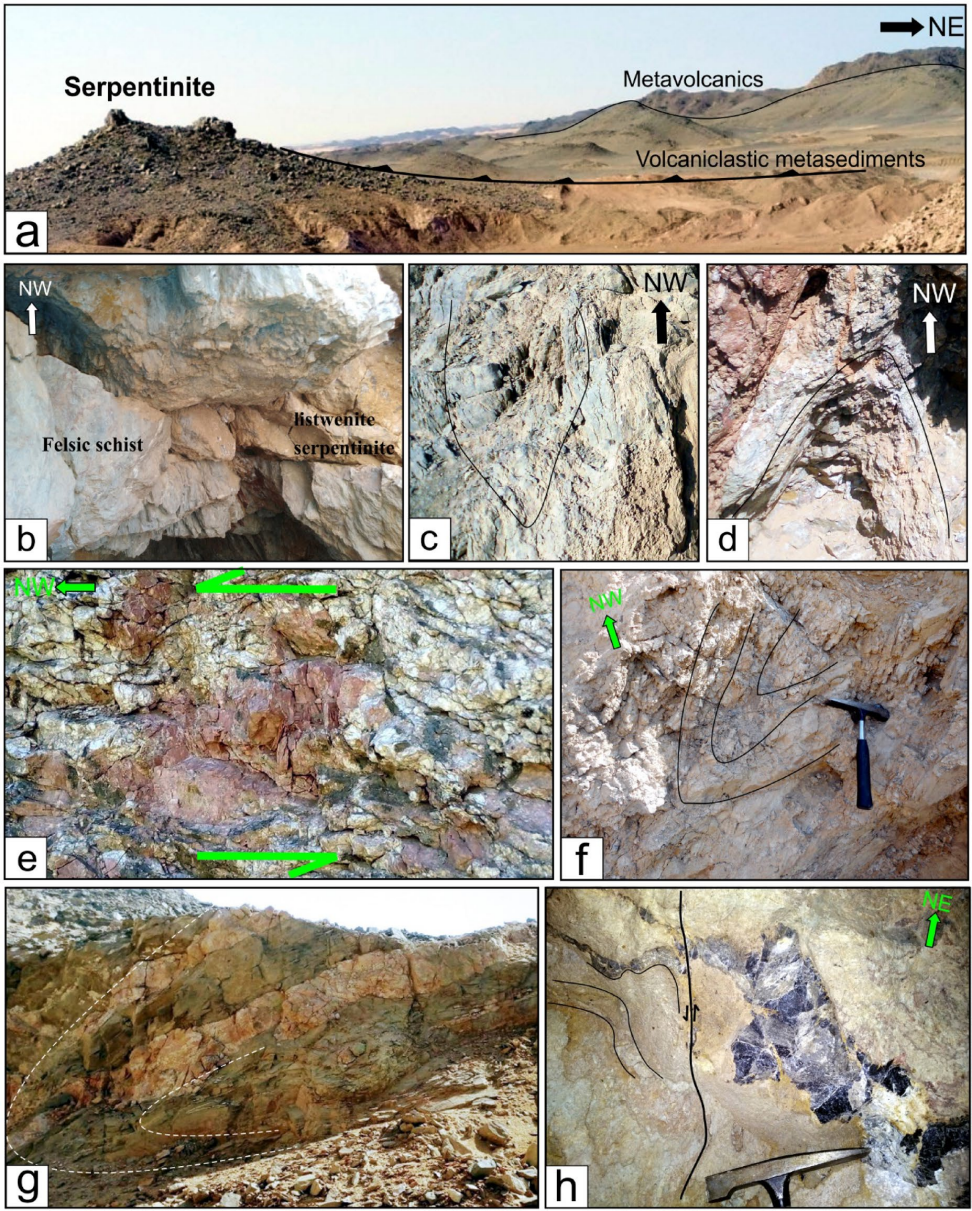
(**a**) Thrust imbrications between ophiolitic slices, volcanoclastic metasediments, and metavolcanics. E-W striking foliation (S1) is preserved within the volcanoclastic metasediments. (**b**) Felsic schist thrusted over listwenite serpentinite, (**c**) NW-trending chevron fold in mafic schist, (**e**) asymmetrical fold in altered schist, (**f**) N-trending overturned fold in altered schist from Haimur gold mine area, (**g**) Overturned fold in amphibolite, (**h**) folded smoky quartz vein along NE-trending axes and displaced by NE-striking sinistral strike slip minor fault.

The N-S shortening phase (D1) in the South Eastern Desert is characterized by an early north–south shortening, resulting from accretion-induced sutures between 750 and 720 Ma^96–98^. This contraction is associated with processes of accretion and obduction involving rocks of the South Eastern Desert terrain^13,26,57,98–102^. The phase occurred between 830 and 720 Ma, coincidentally with the formation of the Allaqi–Heiani arc suture (Fig. 1a and b), which represents the northernmost boundary of the Arabian-Nubian Shield^92,96,98,103^. The western Allaqi shear belt region experienced significant compression during this period, facilitating the formation of E-W foliation within ophiolitic rocks (Fig. 14a) and volcanoclastic metasediments^16^. The S1 structure is oriented WNW-ESE, with a moderate dip directed towards the NNE^16^. The D1 structural features are marked by thrust imbrications that dip initially toward the north and change to NE during D2^16^. The orientation of these thrusts ranges from WNW-ESE to NW–SE (Fig. 14a), effectively defining the interface between the ultramafic rocks of the hanging wall and their highly sheared derivatives found in the footwall, which include amphibolites^16^.

Between 625 and 565 Ma, NE-SW-directed shortening occurred^104,105^, leading to the refolding of nappes with steeply inclined NW-trending folds. Thrust faults between mafic volcanic and felsic schists (Fig. 14b), metavolcanics, and ophiolitic slices mark the D2 phase. The regional S1 planar fabric exhibits significant folding around gently plunging axes oriented to the NW or SE^16^, accompanied by NW–SE striking axial planar foliation (Fig. 14b). Various NW-trending mesoscopic folds (Fig. 14c and d) are present in the volcanoclastic metasediments, felsic and mafic schists, alongside sheared metavolcanics. The mesoscopic and macroscopic folds in the western Allaqi shear belt range from gentle to more tight forms (Fig. 14c and d). The predominant axial planar structure is characterized by crenulation cleavage, with a moderate to steep dip towards the NE or SW. The NW-trending Haseierbah anticline represents the most prominent fold structure in the WASB^16^. In the NW-trending shear zones, mylonites and ultramylonites exhibit a unique mylonitic foliation along with sigmoidal prophyroclasts that indicate a sinistral sense of shearing (Fig. 14e). The N- to NE-dipping thrust faults that formed during the D1 phase, characterized by oblique dip, underwent modifications during the D2 phase^16^.

The E-W compression occurred at 620–580 Ma^14,105,106^. During the D3 phase, early E-W and NW-trending folds underwent deformation into N-trending folds due to dextral shearing along N-S shear zones. The western domain of the western Allaqi shear belt has evolved into a sequence of macroscopic N-trending folds. The strain shifts from a prevailing NE-SW shortening to a more E-W-directed shortening. The overlap of N-trending folds (Fig. 14f) over SW-verging folds creates a crescent-shaped dome interference pattern with distinct serpentinite and gabbro ridges^16^. The most prominent N-trending folds are located at the junction of Wadi Umm Arka and Wadi Tilal Al Qulieb (Fig. 1c).

The western Allaqi shear belt experienced deformation due to NE-trending dextral shear zones (D4), resulting in significant NE-trending folds in the southwestern region. This area primarily comprises folded thrusts within volcanoclastic metasediments, schistose metavolcanics, and ophiolitic materials. The early structural features within this nappe exhibit refolding around NE-trending axes (Fig. 14g). The interaction of NE-SW structures and north-trending folds over earlier northwest-trending folds has formed a prominent crescentic anticline in the southeastern region^16^.

D5 has recent brittle structures from the Early Cretaceous and Red Sea rifting. Strike-slip faults in the western Allaqi shear belt are oriented in ENE-WSW, NE-SW, and N-S directions, disrupting thrust faults and fold axes. These faults are rectilinear, run parallel to F3 and F4 folds, and exhibit a dextral movement (Figs. 1c and 14h) with horizontal displacements ranging from approximately 100 m to 1.5 km. A significant dextral strike-slip fault runs parallel to F4 folds, disrupting the major Haseierbah anticline.

## Mineralization style and lode characteristics

### Haimur Au-deposits

**Fig. 15:**
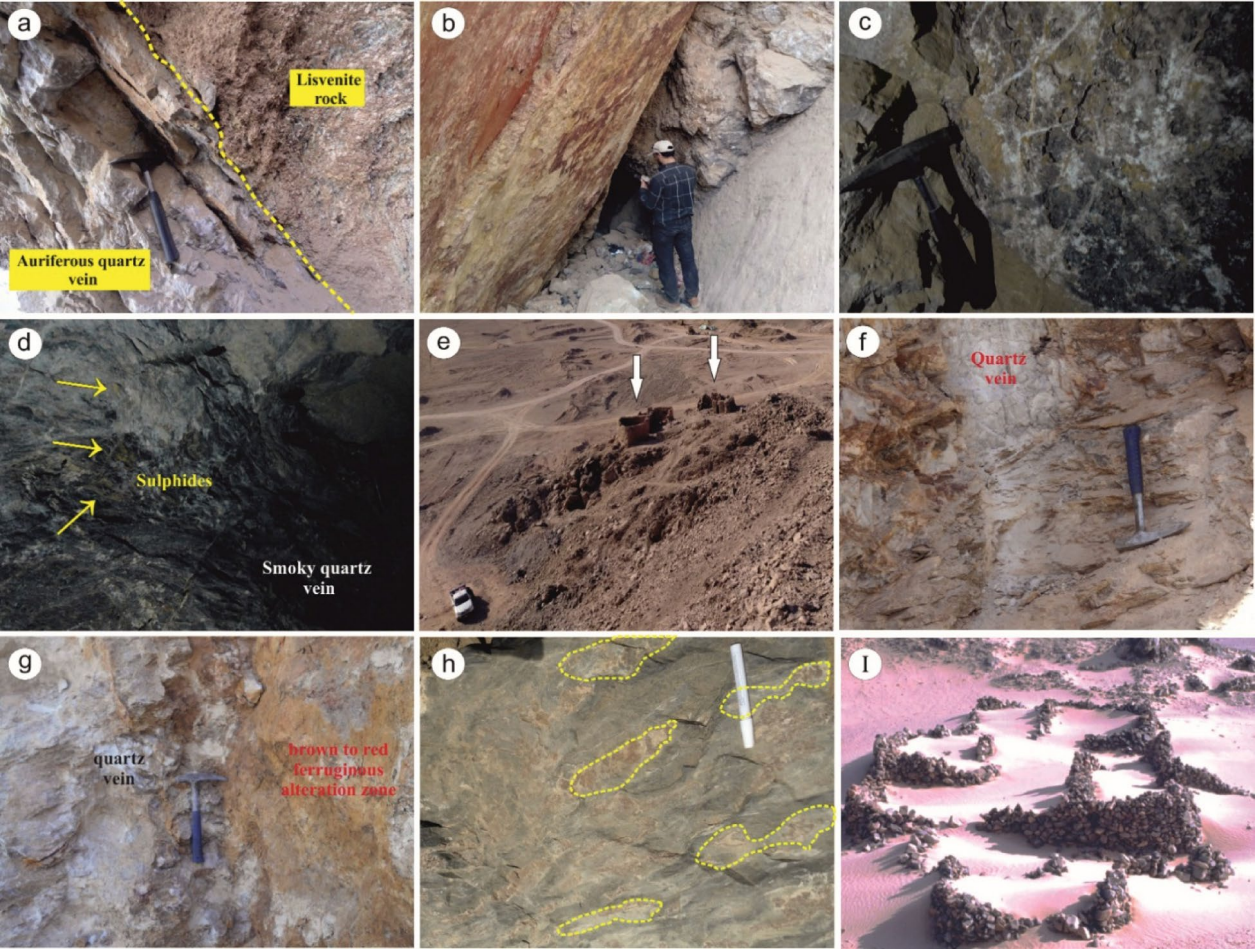
(**a**) Highly-altered listvenite rocks adjacent to the auriferous quartz vein in Haimur deposits, (**b**) Oxidized alteration zones (i.e., ferrugination) surrounding the au-rich quartz vein at Western Allaqi shear belt, (**c**) Stockworks of carbonate veinlets (calcite, dolomite, ankerite) cross-cutting the Au-rich quartz vein in Haimur deposits, (**d**) Scattered batches of visible sulphides within smoky quartz vein at Western Allaqi shear belt, (**e**) Well-preserved ancient stone huts near Haimur gold mine, (**f**) Brecciated-milky-white quartz vein in Um Ashira mineralizing zone, (**g**) Ferruginated alteration zones occurred around auriferous quartz vein in Um Ashira deposits, (**h**) Disconnected lenses and quartz veins showing pinch and swell structural form in Hariari gold deposits, (**i**) Relics of old settlements and ancient buildings near Hariari gold mine^107^.

The Western Allaqi shear belt is located in a region with a highly tectonized and metamorphosed association of pelitic schists, serpentinites, and related listvenite assemblages. Gold mineralization in this area is mainly expressed through a series of smoky/grey (occasionally white) quartz veins, veinlets, and lenses that follow the main shearing direction (NE) and dip at 35°–50°/NW (Fig. 15a). These mineralized quartz veins occupy fractures and cavities within listvenite rocks, which are often sheared and mylonitized, showing enrichment with siliceous components and overprinted by ferruginous alteration (Fig. 15b). The veins are commonly associated with carbonates (calcite, ankerite, and dolomite), along with graphite and visible sulphides, as seen in Fig. 15c and d. Morphologically, these quartz veins and lenses are generally irregular and asymmetrical, with lateral variations in width from a few centimeters up to one meter. However, the average thickness is about 50 cm. Despite their discontinuous nature, individual veins can extend up to 25 m. Evidence of ancient mining activity can be seen in old stone huts, adits, shafts, and surface exposures throughout the ore zones (Fig. 15e).

### Um Ashira Au-deposits

The basement rocks at the Um Ashira area are dominated by gneisses, amphibolites, ophiolitic-island arc associations (schists, metagreywakes, tuffs, marble bands, metagabbros), and granitic intrusions (syn- to late-tectonics). They are dissected by a number of dykes with variable composition from acidic to basic ones^108^. The gold deposits at Um Ashira are confined to brecciated milky to grey quartz veins that cut through gabbroic and granitic host rocks, trending NE and dipping steeply NW. Younger microcrystalline quartz veinlets occasionally crosscut these veins.

The Um Ashira area has numerous holes and small adits referring to ancient mining activities, most of the old workings focused upon the mineralized quartz veins (Fig. 15f) and small areas of alteration zone. These veins have a thickness ranging from a few cm in some places to more than 1 m in others. These wall rock alteration zones exhibit yellowish-brown color due to the Fe-rich alteration (Fig. 15g). Also, they show signs of other alteration processes, including carbonatization and propylitic facies.

### Hariari Au-deposits

The whole area of Hariari is covered by a variety of island arc metavolcanic-metasedimentary assemblage, syn-tectonic granite and late- to post-tectonic intrusions (granite & gabbro), along with post-tectonic dykes and veins^109^. The gold lode in the Hariari area is hosted in multiple mineralized quartz veins embedded within fractures (or cracks). The mineralization zone is within the ENE-trending shear zone, reflecting a remarkable structural control. The auriferous quartz veins are found cross-cutting the hosting gabbroic rocks. They may extend to the neighboring late- to post-tectonic granitoids, which indicates that they were post-dated by the emplacement of the granitic intrusion. The main auriferous vein extends for about 450 m in length and strikes in the ENE direction (dip = 55°/NW). This vein may split into discontinuous quartz lenses extending up to 15 m and 0.5 m as a maximum width. Pinches and swells are detected as a primary structure within the mineralized quartz vein (Fig. 15h). Remnants of old settlements and ancient mining work in the form of mills, washing tables, runners and grinding stones are still preserved in the area near and around the mine (Fig. 15i). The zone around the auriferous veins exhibits a remarkable alteration due to prolonged interaction between the upwelling mineralizing fluids and wallrock. The alteration facies include phyllic, ferruginous, and carbonatization.

## Discussion

Our results, which were obtained from aeromagnetic ED, RS, and field analyses, introduce essential insights into the structural setting controlling the mineralization of the western Allaqi shear belt area. The illustration of alteration zones, shear zones, and deep-seated structures emphasizes the spatial relations between gold occurrences and structural elements. Interpreting these determinations in the context of regional structures and tectonics promotes a more in-depth understanding of mineral potential strategies.

### Lithological mapping

The differences in tonal characteristics among the identified color composites, specifically Decorrelated FCC 657, MNF324, and PCs 132 and 342 in RGB, within the specified area of interest, were instrumental in delineating lithological boundaries and enabled the examination of significant structural features, such as thrusts, strike-slip faults, and folds. This process significantly contributed to the refinement of geological and structural maps. The colored imagery provided a means to trace major foliation, thereby assisting in the identification of prominent syncline and anticline structures within the study area. The thrust contacts between the ophiolitic sequence and arc assemblages were effectively highlighted and delineated using the designated color composites based on their tonal variations. Furthermore, the sharp contacts between syn- and post-magmatic sequences and other rock units in the area were also accentuated. Among these, PC 132 proved to be the most effective color composite for distinguishing lithological boundaries between serpentinites-talc carbonates, arc metavolcanics, and volcanoclastic metasediments (Fig. 4c). Additionally, distinguish between weathered and massive syn/post granitoids. The Decorrelated FCC 657 (Fig. 4a) facilitated the detection of post-massive granite. This lithological discrimination enabled the identification of favorable rock compositions within the delineated alteration zones throughout the study area.

### Hydrothermal alteration zones mapping

The band ratio indices of 6/4 (indicative of ferrous iron oxides), 6/5 (representing ferric oxides), 6/7 (associated with hydroxyl-bearing alterations), and 7/5 (representing the chlorite zones) serve as crucial indicators for the presence of Fe3 + , Fe2 + , Al/Fe-OH, Mg-Fe-OH, and Si–OH groups, as identified through the spectral bands of Landsat-8^61^. These ratios were utilized to due to their diagnostic absorption within the wavelength ranges of 0.45–0.52 µm, 0.845–0.885 µm, and 2.08–2.35 µm corresponding to bands 4, 5, 6, and 7 of the Landsat-8 satellite.

In BR 6/7 (Fig. 5a), the hydroxyl-bearing alterations were recorded along areas of serpentinites, talc carbonates, volcanoclastic metasediments, and syn-tectonic metagabbro. Meanwhile, the ratio of 7/5 was revealed in all the regions enriched by chlorites, which were recorded within the arc metavolcanics, including felsic to intermediate metavolcanics and volcanoclastic metasediments (Fig. 5b). Both ratios of 6/4 and 6/5 (Fig. 5c and 5) successfully recorded the ferrous and ferric-bearing minerals zones, which are recognized over the areas of mainly metavolcanic composition (e.g., felsic to intermediate metavolcanics and volcanoclastic metasediments), as well as scattered parts over both syn-tectonic metagabbro and granitoids. The four detected zones of hydroxyl-bearing alterations, chlorite zones, ferrous and ferric bearing minerals zones were stacked as one map reflecting the general spatial distribution of the major shear zones which were allocated via Landsat-8 (Fig. 7a). This surface distribution of the altered areas using Landsat-8 were coincided with the plotted sites of the old gold mines which reflect the accuracy of the detected alteration zones (Fig. 7a).

**Fig. 16:**
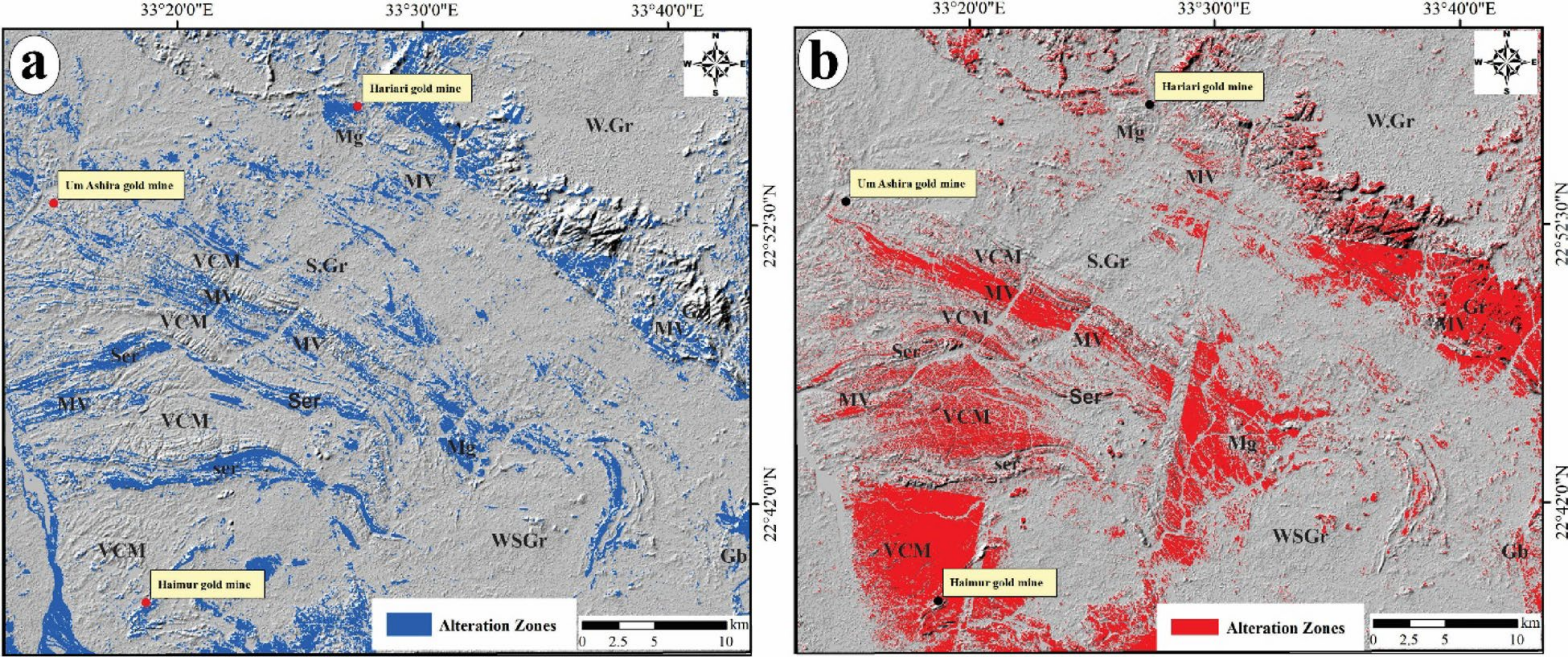
General surface distribution of all detected hydrothermal alteration zones via (**a**) Landsat-8 and (**b**) ASTER data. For abbreviation, see Fig. 4.

Along with the BRs of Landsat-8, the implementation of CEM for the supervised classification of ASTER data is effective in identifying the spatial distribution of thirteen key alteration minerals, which are representative of four typical alteration zones, namely argillic, phyllic, propylitic, and gossan zones (Fig. 6a–d). The spatial distribution of the argillic zones was aligned mainly with the areas of volcanoclastic metasediments, metavolcanics and syn-tectonic granites (Fig. 6a), while the pyllic zones covered the areas of post-tectonic granites besides the aforementioned lithological units which covered by the argillic zones (Fig. 6b). The propylitic zones were covered broad areas over the investigated areas which represented the major southern sector of the study area of prevalent volcanoclastic metasediments compositions as well as scattered parts along the serpentinites, metavolcanics, post gabbro and weathered syn and post granites (Fig. 6c). Gossan zones were more concentrated at the southwestern sectors and along the volcanoclastic metasediments belts striking NW at the northeastern part of the study area (Fig. 6d). The four alteration zones were combined together as a whole alteration zones map which displays the surface distribution of the alteration zones detected by ASTER data (Fig. 16b). According to Fig. 16a, the spatial distribution of these altered zones was aligned with the same altered areas which recognized utilizing band ratios of Landsat-8 as well as the ASTER alteration zones were allocated somewhat at the same gold sites in the study area (Fig. 16b).

### Integration of hydrothermal alteration mapping and lineament density

The significance of hydrothermal alteration zones as prime environments for the genesis and existence of ore deposits is well established. As a result, identifying and mapping these zones are vital for locating areas likely to contain corresponding ore deposits, particularly gold deposits in the study area. Optical data from Landsat-8 and ASTER have been utilized to aid in this endeavor. Hydrothermal minerals, including carbonates and those rich in Fe and Mg-OH, within alteration zones indicates the alteration processes impacting basic-ultrabasic and granitic rock formations. These minerals display distinct spectral properties, as documented by^110^.

**Fig. 17:**
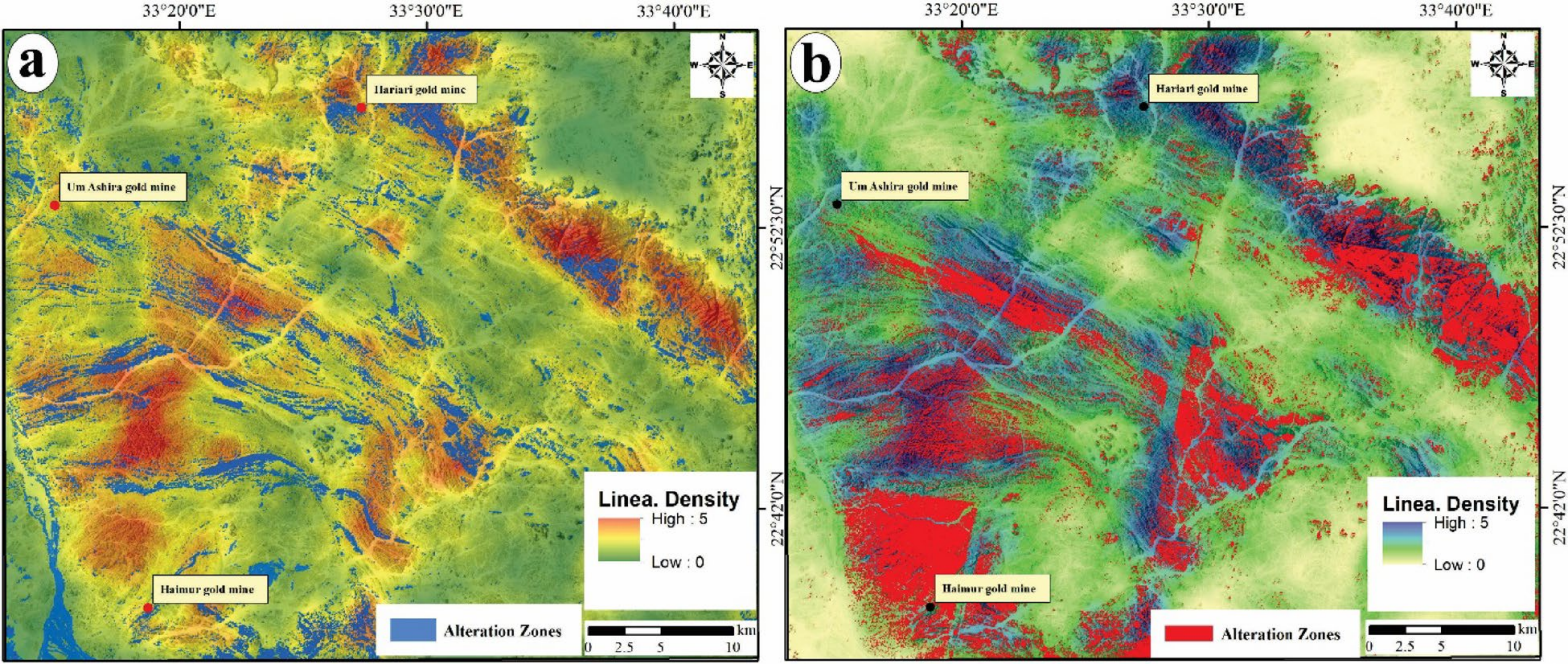
Potential maps highlight the lineament density against the spatial distribution of the allocated alteration areas over the study area by (**a**) Landsat-8 and (**b**) ASTER.

An elevated density of lineaments may imply a greater extent of rock fracturing^84^, which is commonly linked to mineralization processes. Lineaments and fractures act as vital channels for the movement of hydrothermal solution fluids. The potential for gold mineralization is amplified in regions with a considerable density of lineaments and fractures associated with hydrothermal alteration zones. Consequently, the lineament density map (Fig. 7b) has been layered over the alteration zones (Fig. 16a and b), which have been accurately characterized using Landsat-8 band ratios and the CEM method applied to ASTER data. The spatial correlation between the alteration zones and the surface distribution of lineament density (Fig. 17a and b), which reveals exceptionally high lineament densities, emphasizes the critical role of these features in the mineralization process. A detailed analysis of this map may uncover the most probable mineralization zones within the study area.

The observed distribution of lineament density and surface alteration zones has created two potential maps (Fig. 17a and b) for the specified regions, illustrating their interrelations and identifying areas with a heightened likelihood of ore deposits associated with alteration minerals. The analysis indicates that the recognized alteration zones are predominantly located in regions exhibiting moderate to high lineament density, characterized by serpentinites, metavolcanics, volcaniclastic metasediments, and syn-tectonic metagabbro and granodiorite. These geological features facilitate the effective movement of hydrothermal fluids and the subsequent deposition of ores. Nevertheless, certain areas within the study region exhibit high lineament density but lack alteration minerals or zones. This finding suggests that the lack of alteration may be attributed to variations in rock composition or that these high-density lineament regions were not significantly affected by hydrothermal fluids, leading to the absence of alteration minerals. Furthermore, it has been previously noted that the predominant orientation of these lineaments is primarily NE to NNE and N-S. Consequently, it can be deduced that these tectonic orientations have shaped and influenced most lineaments, which are considered favorable conduits for ore-bearing fluids in the study area.

### Aeromagnetic analysis

Advanced ED approaches to RTP data and UWC-RTP at different depths demonstrate critical tectonic and structural controls within the studied provinces^5,9,40^. The HTF and NED techniques reveal superior precision in outlining geological boundaries/contacts and faults without producing incorrect edges, providing a more trustworthy structural interpretation^45,46^.

The HTF and NED results of western Allaqi shear belt at various depths demonstrate a systematic variation in structural directions. HTF and NED of RTP (Figs. 10, 11, and 12) showed that the WNW is dominant in western and central parts of the study area^64,65,111,112^, while the N-S is the prevailing structure in the eastern side^113,114^. At depths of 0.5 km, 1 km, and 1.50 km, the dominant trends are WNW, NE, E-W, NW, and ENE, while at deeper altitudes (3 km), the WNW, E-W, ENE, NNE, NE, and NW become more recognized. Notably, the N-S structural direction reduces with depth, whereas the E-W direction becomes prevalent, which may indicate a transformation in structural effect at various crustal levels. This interpretation suggests that shallow structural networks are more affected by recent deformation processes, while deeper ones maintain more aged tectonic patterns.

Comparing these results with earlier investigations, comparable directions have been recognized in structurally complicated provinces of the Eastern Desert. For example, Eldosouky et al.documented that the ENE, NNE, and NW directions control shallow altitudes. At the same time, deeper faults align with E-W and N-S trends predominantly in the eastern parts of the area, revealing tectonic compartmentalization due to protracted tectonic events. Besides, Sehsah et al.^87^ noted WNW and NNE dominate western parts while the N-S and NNE fault systems are the prevailing deep-seated at eastern parts of the Allaqi-Heiani Suture^52,57,101^, which were overprinted by post-accretionary strike-slip faults at shallow depths.

### Modes of deformation characterizing the western Allaqi shear belt

In the western Allaqi shear belt, the D1 phase is characterized by the formation of an axial planar cleavage (S1) that strikes east–west and dips steeply to the north, which is associated with tight to isoclinal folds that plunge to the east. In the vicinity of Wadi Haimur, D1 is exemplified by shear zones related to thrusting that occurred during the emplacement of mafic and ultramafic rocks from ophiolitic assemblages over the volcanic and sedimentary deposits of the island arc. The D2 phase introduces folds that affect the thrust surfaces, oriented east–west with hinges that plunge westward and possess steeply dipping axial planar cleavages to the northeast and east–northeast^16^. D2 is also marked by shear zones trending west-northwest to east–southeast and northwest to southeast, varying in width from several meters to as much as 3 km, featuring sharp and gradual boundaries that encircle lenses of less deformed rock. The D3 phase in the western Allaqi shear belt has transformed the earlier east–west and northwest-trending folds into north-trending folds due to dextral shearing along north–south shear zones^16^. This modification reshaped the structural elements of the western domain into macroscopic N-trending folds. The strain transitioned from predominant N-S shortening to more E-W-directed shortening, resulting in N-trending upright folds, steeply dipping axial planar foliation, and NE-trending sinistral strike-slip faults. During D4, the western Allaqi shear belt experienced deformation due to NE-trending dextral shear zones, resulting in significant NE-trending folds in the southwestern region. This area is composed of folded thrusts within volcanoclastic metasediments, schistose metavolcanics, and ophiolitic materials. The interaction of NE-SW structures and north-trending folds over earlier northwest-trending folds formed a prominent crescentic anticline^16^ in the southeastern portion of the study area. During D5, the western Allaqi shear belt has strike-slip faults oriented in ENE-WSW, NE-SW, and N-S directions, disrupting thrust faults and fold axes. These faults are rectilinear, parallel to F3 and F4 folds, and have a sinistral movement^16^.

Sinistral strike-slip shearing correlates with the elongation of clasts sourced from felsic pyroclastic rocks and NW-trending folds. The subsequent east–west (E–W) shortening is attributed to a post-accretion phase, which is probably connected to the concluding stages of the Pan-African orogeny or the Najd orogeny^13,16,26,56,97,101,115,116^.

During D5, the belt exhibits dextral strike-slip faults oriented in the ENE-WSW, NE-SW, and N-S directions, disrupting thrust faults and fold axes^16^. A significant dextral strike-slip fault-oriented ENE-WSW in the southeastern part disrupts the major Haseierbah anticline, minor folds, and N-S sinistral strike-slip faults.

D4 structures control the gold deposits in WASB. D4 is marked by S4 axial planar foliation, NE-trending folds, and dextral shearing. In the Haimur Au-deposits, quartz veins, veinlets, and lenses align with the primary shearing direction (NE-SW). In the Um Ashira Au-deposits, brecciated milky to grey quartz veins intersect gabbroic and granitic host rocks, trending NE and dipping steeply to the NW. In the Hariari Au-deposits, the principal auriferous vein stretches approximately 450 m long and strikes in the ENE direction.

## Conclusions

- Ophiolitic and island arc rock formations characterize the western Allaqi shear belt. The ophiolitic suite includes serpentinite, talc carbonate, listwenite, metagabbro/amphibolite, and metabasalt, all with a tholeiitic composition. Listwenite is produced through carbonation and silicification processes linked to hydrothermal activity. Island arc rocks are a mix of volcanoclastic metasediments and metavolcanics, formed during the early immature stage of an island arc tectonic setting.
- Advanced ED approaches (HTF and NED) were applied to RTP and UWC data of western Allaqi shear belts to delineate shallow and deep structures. HTF and NED filters of RTP data showed that the N-S to NW directions are the dominant trends. At depths of 0.5, 1, and 1.5 km, the prevailing trend is the WNW direction with N-S, NE, NW, and E-W orientations. While at a depth of 3 km, the WNW, E-W, ENE, NE, NNE, and NW are the dominant directions in the western Allaqi shear belt. Notably, the WNW prevails in the western and central regions, while the N-S is predominant in the eastern part of the area.
- The study uses Landsat-8 spectral bands to identify minerals in a region, including ferrous iron oxides, ferric oxides, hydroxyl-bearing alterations, and chlorite zones. The findings are stacked to reflect major shear zones. CEM for supervised classification of ASTER data identified thirteen key alteration minerals, aligning with the spatial distribution of these altered zones.
- Hydrothermal alteration zones are crucial for the genesis and existence of ore deposits, particularly gold. Identifying and mapping these zones is essential for locating areas with corresponding ore deposits, particularly gold. Optical data from Landsat-8 and ASTER has been used to study these zones. The presence of hydrothermal minerals, such as carbonates and those rich in Fe and Mg-OH, within alteration zones indicates alteration processes impacting basic-ultrabasic and granitic rock formations. Elevated lineament density may indicate greater rock fracturing, which is linked to mineralization processes. Lineaments and fractures act as channels for hydrothermal solution fluid movement, and the potential for gold mineralization is amplified in regions with high lineament densities. Two potential maps have been created to identify areas with a heightened likelihood of ore deposits associated with alteration minerals.
- The western Allaqi shear belt exhibits multiple phases, specifically D1 through D5. The D1 phase is marked by forming a foliation (S1) that strikes east–west and dips steeply to the north, accompanied by tight to isoclinal folds that plunge to the east. In the D2 phase, folds alter thrust surfaces, characterized by hinges that plunge to the west and steeply dipping axial planar cleavages oriented northeast to east–northeast. The D3 phase further modifies the earlier E-W and NW-trending folds into north-trending configurations, a result of dextral shearing along north–south shear zones, thereby reshaping the structural characteristics of the western domain into prominent north-trending folds.
- During D4, the western Allaqi shear belt experienced deformation due to NE-trending dextral shear zones, resulting in significant NE-trending folds in the southwestern region. This area is composed of folded thrusts within volcanoclastic metasediments, schistose metavolcanics, and ophiolitic materials. During D5, the western Allaqi shear belt has strike-slip faults oriented in ENE-WSW, NE-SW, and N-S directions, disrupting thrust faults and fold axes.
- Structural investigations reveal polyphase deformations (D1-D5), characterized by S1 foliation and regional thrusting. Field observations show F1 folds as shear pods inside major F2 fold hinges. In D2, sinistral strike-slip shearing, characterized by a predominant thrust component, is observed in shear planes. Evidence for this shearing includes sub-horizontal stretching lineations, elongated clasts from felsic pyroclastic rocks, and the formation of NW-trending folds.
- The Haimur gold deposits are expressed by smoky/grey quartz veins, veinlets, and lenses. The mineralized quartz veins occupy fractures within listvenite rocks following the main course of shearing (NE-direction) and dipping 35°-50°/NW, often sheared and mylonitized. Au-rich quartz veins are associated with carbonates and sulphides. The quartz veins are irregular and unsymmetrical, with lateral variation.
- The Um Ashira Gold deposits are confined to highly-brecciated quartz veins, which cut through the rocks. These veins are trending in NE direction and commonly steeply dipping to NW. The area has numerous holes and small adits indicating ancient mining activities, focusing on mineralized quartz veins and alteration zones. These zones exhibit yellowish-brown color due to Fe-rich alteration and have been subjected to other alteration processes.
- The gold-lode in Hariari is linked to multiple-mineralized quartz veins embedded within fractures. The mineralization zone is located within the ENE-trending shear zone, with auriferous quartz veins found cross-cutting gabbroic rocks and possibly extending to neighboring granitoids. Relics of old settlements and mining work are still present.

## Data Availability

Data sets generated during the current study are available from the corresponding author on reasonable request.
